# Key Components and Barriers in Web-Based Suicide Prevention Gatekeeper Training: Systematic Narrative Review

**DOI:** 10.2196/81572

**Published:** 2026-02-05

**Authors:** Olivier Ferlatte, Emmanuelle Gareau, Keven Lee, Kinda Wassef, John Lindsay Oliffe, Hannah Kia, Brock Dumville

**Affiliations:** 1 École de santé publique de l’Université de Montréal Montréal, QC Canada; 2 Centre de recherche en santé publique Montréal, QC Canada; 3 School of Nursing University of British Columbia Vancouver, BC Canada; 4 Department of Nursing University of Melbourne Melbourne Australia; 5 School of Social Work University of British Columbia Vancouver, QC Canada; 6 Suicide Prevention Centre of Montreal Montréal, QC Canada

**Keywords:** narrative review, suicide prevention, suicide, online, web-based, gatekeeper training

## Abstract

**Background:**

Gatekeeper training programs (GTPs) are a key component of contemporary suicide prevention strategies, equipping community members and non–mental health professionals with the skills to identify, engage with, and refer individuals at risk of suicide. Increasingly, these programs are delivered via the web, offering a compelling alternative to in-person training through greater scalability, flexibility, and cost-effectiveness. However, little consensus exists regarding the design, modes of delivery, and implementation strategies of web-based GTPs. Further, there is a limited understanding of which components affect their usability and engagement.

**Objective:**

This systematic narrative review aims to identify the key components—including facilitators and barriers—of web-based GTPs.

**Methods:**

We systematically searched web-based databases (CINAHL, Embase, MEDLINE, PsycINFO, and Web of Science) to identify peer-reviewed articles published between 2000 and 2025 that involved web-based GTPs. After screening, 59 studies met the inclusion criteria and were analyzed using content analysis to identify key components and barriers affecting the delivery and receipt of web-based GTPs.

**Results:**

Results were organized under 3 categories: design, content, and pedagogy. Key design considerations emphasized accessibility for diverse learning styles and digital literacy levels, customizability for different user groups, privacy protection, and the long-term sustainability of training content and delivery platforms. Core training content covered four domains: (1) suicide-related knowledge (eg, prevalence, myths, and at-risk groups), (2) gatekeeping skills (eg, understanding risk factors, recognizing warning signs, problem-solving and safety planning), (3) resource awareness (eg, available local resources and referral procedures), and (4) general mental health education (eg, mental fitness, mindfulness, and self-care strategies for gatekeepers). In terms of pedagogy, the reviewed studies used a wide range of strategies that comprised interactive learning activities (eg, simulation, practice exercises), periodic knowledge checks (eg, quizzes), and reinforcement mechanisms (eg, booster sessions). Additionally, fostering a sense of community (eg, online support spaces or discussion forums) and promoting trainees’ autonomy (eg, self-paced training) were highlighted as key components of training delivery.

**Conclusions:**

Web-based GTPs represent a promising avenue for expanding access to suicide prevention training. Their effectiveness may be strengthened through the integration of frameworks tailored to web-based learning environments, as well as interactive and user-centered design elements that support learning and retention. Future research should examine the acceptability, feasibility, and sustainability of these programs, while also refining their adaptation for diverse populations. In this regard, co-design approaches could facilitate the tailoring of such programs to the needs and specificities of their target populations. Overall, enhancing the design and delivery of web-based GTPs may ultimately improve their contribution to suicide prevention efforts.

## Introduction

Suicide is a critical public health issue worldwide, with significant social, emotional, and economic impacts on individuals, families, and communities [1]. As one of the leading causes of preventable death [2], suicide requires multifaceted approaches including awareness raising, reducing mental illness stigmas, and enhancing timely interventions [3]. Among these strategies, gatekeeper training programs (GTPs) have emerged as a cornerstone in equipping individuals to identify, approach, and support those at risk of suicide [4]. Gatekeepers are non–mental health professionals who may have contact with individuals at risk of suicide (ie, educators, parents, peers, or other community members), and are trained to recognize warning signs, initiate conversations about suicide, and connect individuals to appropriate professional help [1,5]. These programs, which are endorsed by the World Health Organization (WHO) [6], have demonstrated effectiveness in various settings and populations [7], highlighting their potential key role in suicide prevention.

Traditionally, GTPs have been delivered through in-person workshops and seminars [5], offering opportunities for direct interaction, role-playing, and immediate feedback. However, advances in technology and the increasing digitization of health education have led to the development and adoption of web-based GTPs [8,9]. Web-based formats provide significant advantages, including scalability, accessibility for geographically dispersed participants, and the ability to tailor training to diverse populations [7,10,11]. These programs can not only overcome logistical barriers (eg, physical attendance or availability of training) [12] but can also be particularly valuable for populations where confidentiality and anonymity are essential, such as stigmatized communities, including migrants and lesbian, gay, bisexual, trans, queer, and other sexual and gender minorities (LGBTQ+) populations [13]. Indeed, confidentiality can encourage more meaningful engagement with sensitive topics such as mental health and suicide among communities already affected by cumulative stigmas [14]. In addition, web-based training allows participants to learn at their own pace, accommodating busy schedules and varying levels of prior knowledge [15].

Recent evaluations indicate that web-based and in-person GTPs have similar effectiveness [8,9,11,14]. However, there are implementation challenges for web-based programs, including limited internet access, technological difficulties, varying levels of digital literacy, and user engagement issues [15]. Moreover, the design and content of web-based training are critical, and poorly structured or overly generic programs may fail to meet the complex and varied needs of participants [16].

Nonetheless, recent advancements in interactive technologies present unique opportunities to innovate and scale suicide prevention with GTPs. However, effectively harnessing web-based technologies requires a clear understanding of both the factors that contribute to program success and the challenges that hinder implementation. This systematic narrative review synthesizes the current evidence for web-based GTPs, addressing two key questions: (1) What are the key components of promising and successful web-based suicide prevention GTPs? (2) What are the barriers to delivery and usability of these programs? In summarizing the existing literature, this review provides recommendations for the development, implementation, and scaling of web-based GTPs, with the overall goal of contributing to global efforts for reducing suicide.

## Methods

### Overview

This systematic narrative review was conducted following the PRISMA (Preferred Reporting Items for Systematic Reviews and Meta-Analyses) guidelines (Multimedia Appendix 1) [17] and has been registered with PROSPERO (Registration ID: CRD42023462414). Since this study is a review of published, peer-reviewed articles, ethical approval was not required.

### Search Strategy

A comprehensive search strategy was developed and conducted under the guidance of a specialized university librarian. The strategy combined 3 main concepts: suicide prevention, gatekeeping intervention, and web-based training. The full search strategy is available in Multimedia Appendix 2. The search was initially launched on October 1, 2023, and conducted across 5 databases: CINAHL, Embase, MEDLINE, PsycINFO, and Web of Science. Additionally, we searched the first 20 pages of Google Scholar, as results beyond the first 20 pages were not related to our 3 main concepts. The reference lists of all subsequently included articles were manually reviewed to identify further relevant sources. To ensure the inclusion of the most recent literature, the complete search strategy was relaunched on June 19, 2025.

### Eligibility Criteria

We included peer-reviewed research articles on web-based GTPs, published in English or in French from 2000 onwards. We included all types of studies and designs, except for articles that did not discuss the web-based components of the training and research protocols. Articles focusing on general mental health programs not specific to suicide prevention were excluded. In addition, articles targeting mental health professionals (eg, psychotherapists, psychiatrists, and mental health nurse practitioners) were excluded, as their educational background and professional experiences could result in significantly different training components, facilitators, and barriers compared with training programs for laypersons [18]. Articles targeting health care professionals outside specialized mental health—such as pharmacists, pharmacy staff, health care lecturers, and police officers—were eligible for inclusion. Studies published before 2000 were also excluded due to significant technological advancements since that time.

### Screening and Study Selection

Using Covidence, EG, KW, and KL independently screened all titles and abstracts. Any records deemed potentially relevant by at least one reviewer were then subjected to a full-text examination, which followed the same independent screening process. In case of disagreement after full-text screening, EG, KW, and KL discussed their interpretations of the articles until they reached consensus. If consensus could not be achieved, OF served as a third-party mediator.

### Data Extraction

Data extraction was conducted using Covidence. KW and EG independently extracted the following descriptive data items:

1. Methodological information: Geographical context, study goals, research approach, and study design.
2. Gatekeeper training program information: Program name, training setting, format, if training was adapted from an existing program, development process, training supporting platform, training objectives, training description, organizing institution, trainees’ characteristics, number of trainees, components of training, topics covered by training, effectiveness of the training, target population of the gatekeeping intervention.

Conflicts in data extraction were resolved jointly by KW and EG using the Covidence Consensus tool. These data were used to characterize the included studies and inform Table 1, but were not analyzed further in the identification of barriers or facilitators. The analysis of facilitators and barriers was conducted independently of the data extraction process and is detailed in the following section.

**Table 1:** A brief overview of the characteristics of the training programs included in the systematic narrative review of web-based gatekeeper training programs (GTPs).

Author	Name	Components of training	Topics covered^a,b^				
			Information about suicide	Information about being a gatekeeper	Information about resources/referrals		Information about general mental health
Afsharnejad et al [19]	Talk-to-me MOOC^c^	Videos, quiz/tests	✓	✓			
Albritton et al [20]	Be present	Videos, e-tool box, homework assignments, social media posting	✓				
Bartgis and Albright [21]	Kognito gatekeeper simulations	Role-play/simulation		✓	✓		
Brown et al [22]	Indigenous network suicide intervention skills training (INSIST) program	N/S^d^	N/S	N/S	N/S		N/S
Bryant et al [23]	Kogito “At Risk Primary Care”	Role-play/simulation		✓	✓		
Canady [24]	Signs matter: early detection	Role-play/simulation quiz/tests, resources	✓				
Canady [24]	At-risk for high school educators	Role-play/simulation quiz/tests, resources	✓	✓	✓		
Canady [24]	At-risk for middle school educators	Role-play/simulation quiz/tests, resources	✓	✓	✓		
Carpenter et al [25]	Online veteran administration’s (VA) suicide prevention GTP (S.A.V.E./SAVE [signs, ask, validate, encourage/expedite])	Videos, role-play/simulation	✓	✓			
Caughlan et al [26]	Mind4Health	Videos, readings, resources		✓	✓		
Cohen et al [27]	Israeli gatekeeper training	Role-play/simulation	✓		✓		
Colder Carras et al [28]	Stack-Up overwatch program	N/S	N/S	N/S	N/S		N/S
Coleman et al [29]	Kognito at risk for college students	Role-play/simulation		✓	✓		
Colucci et al [30]	Suicide first aid guidelines training	Videos, quiz/tests, infographics, homework assignments, reflective journaling	✓	✓	✓		
Ghoncheh et al [8]	MHO	PowerPoint presentation, audio features, graphs, quiz/tests, reading material, discussion board	✓	✓	✓		
Ghoncheh et al [8]	Children and family court advisory and support service program	N/S	N/S	N/S	N/S		N/S
Ghoncheh et al [8]	Question persuade and respond (QPR) online gatekeeper training	PowerPoint presentation, role-play/simulation, quiz/tests, reading material, videos, audio features	N/S	N/S	N/S		N/S
Ghoncheh et al [8]	Hollywood homeless youth partnership (HHYP) program	PowerPoint presentation, audio features, quiz/tests	N/S	N/S	N/S		N/S
Ghoncheh et al [8]	In the line of duty	Videos, audio features	N/S	N/S	N/S		N/S
Ghoncheh et al [15]	MHO	PowerPoint presentation, quiz/tests, discussion board	✓	✓	✓		
Hawley et al [31]	Not applicable	Didactic content, video and audio clips, and reflection questions	✓	✓	✓		✓
Hill and McCray [32]	The Texas ask about suicide to save a life (AS + K?) suicide GTP	N/S	✓	✓	✓		
Hill et al [33]	ASK about suicide to save a life (AS + K?)	Videos	✓	✓			
Hofmann et al [34]	COPS (coping with suicide)	Videos, reading material, worksheets, quiz/tests	✓	✓	✓		
Hofmann and Wagner [35]	N/S	Videos, audio plays, manual	✓	✓	✓		✓
Holmes et al [14]	Start	N/S	N/S	N/S	N/S		N/S
Kawashima et al [36]	N/S	PowerPoint presentation, videos	✓	✓	✓		
Kimbrel et al [37]	Safety planning intervention (SPI)	PowerPoint presentation, videos, role-play/simulation, reading material, worksheets		✓			
Kingi-Ulu’av et al [38]	LifeKeepers booster session	Readings	✓	✓	✓		
Kingi-Ulu’av et al [7]	QPR	N/S	N/S	N/S	N/S		N/S
Kingi-Ulu’av et al [7]	MHO	N/S	N/S	N/S	N/S		N/S
Kingi-Ulu’av et al [7]	I CARE	N/S	N/S	N/S	N/S		N/S
Kingi-Ulu’av et al [7]	Act on FACTS: making educators partners (MEP)	N/S	N/S	N/S	N/S		N/S
Kingi-Ulu’av et al [7]	Kognito gatekeeper simulations	N/S	N/S	N/S	N/S		N/S
Kreuze and Ruggiero [39]	Kognito at-risk for high school educators	Videos, role-play/simulation		✓	✓		
Kreuze and Ruggiero [39]	QPR	Videos	✓	✓	✓		
Kreuze and Ruggiero [39]	MEP in youth suicide prevention: ACT on FACTS	Videos, role-play/simulation, audio features	✓	✓	✓		
Kreuze et al [10]	QPR	Videos, testimonials, narration, bulleted lists, mnemonics, pocket cards, role-play/simulation, self‐audit checklist	✓	✓	✓		
Kreuze et al [10]	MEP in youth suicide prevention	Videos lectures, expert content, conversations example, role-play, testimonies, activities related to videos	✓	✓	✓		
Lamis et al [40]	MEP in youth suicide prevention: ACT on FACTS	Lecture, question and answers, digital vignettes/interactive activities	✓	✓	✓		
Lancaster et al [9]	Web-based QPR	Videos, text, pictures, audio features	N/S	N/S	N/S		N/S
Lee-Tauler et al [41]	Chaplains-CARE online program	Didactic lectures, videos, reading material, quiz/tests, interactive activities	✓	✓			✓
Liu et al [42]^e^	Various	Various	N/S				
MacDonald Hart et al [43]	Suicide intervention first aid (SIFA)	Didactic lectures, interactive, discussions, role-play/simulation exercise, skills practice	✓	✓	✓		
Manning and Van Deusen [44]	Western Michigan University suicide prevention program online course	Videos, photographs, and graphics	✓	✓	✓		
Marley et al [45]	Pharm-SAVES training	N/S		✓	✓		
McKay et al [46]	Living works start	Videos, reading material	✓	✓	✓		
Mirick [47]	SOS (signs of suicide) for school staff	Role-play/simulation with both child and adolescent	✓	✓	✓		
Mishkind et al [48]	VitalCog: suicide prevention in the workplace (formerly known as Working Minds)	Videos, role-play/simulation, group discussion, workbook	✓	✓	✓		
Osteen et al [49]	QPR for law enforcement	N/S		✓			
Perepezko et al [50]	Stack-Up overwatch program	N/S	N/S	N/S	N/S		N/S
Pilbrow et al [51]	Advanced suicide prevention training for pharmacists	Video, role-play/simulation, digital workbook, group discussion	✓	✓	✓		
Postuvan et al [52]	IAlive (iˇZiv in Slovenian)	Videos lectures, animated examples, interactive images/graphics with pop-ups	✓	✓			
Quinnett [53]	QPR pathfinder training	Video, role-play/simulation, reading	✓	✓	✓		✓
Reifegerste et al [54]	Help for relatives	Videos, audio-recordings, manual, text content	✓	✓	✓		✓
Rein et al [55]	Kognito	Role-play/simulation		✓			
Robinson-Link et al [56]	Kognito	Role-play/simulation	✓	✓	✓		
Roslan et al [57]	Online advanced C.A.R.E suicide prevention GTP (AdCARE)	Role-play/simulation	N/S	N/S	N/S		N/S
Ross et al [58]	Suicide prevention for college student gatekeepers program	Role-play/simulation, skills practice, discussions, peer cofacilitation	✓	✓			
Ross et al [59]	Suicide prevention for college student gatekeepers program	Role-play/simulation, skills practice, discussions	✓	✓			
Schmeckenbecher et al^e^ [60]	Various	N/S	N/S	N/S	N/S		N/S
Seabury [61]	Crisis counseling: I Am Chipper!	Interactive PowerPoint presentation, videos, role-play/simulation, reading material	✓	✓			
Seabury [61]	Suicide assessment: rube farmer	Interactive PowerPoint presentation, videos, role-play/simulation, reading material	✓	✓			
Seabury [62]	Crisis counseling: I Am Chipper!	Interactive PowerPoint presentation, videos, role-play/simulation, reading material, quiz/tests	✓	✓			
Seabury [62]	Suicide assessment: rube farmer	Interactive PowerPoint presentation, videos, role-play/simulation, reading material, quiz/tests	✓	✓			
ShantaBridges et al [63]	Suicide prevention and awareness for depression	N/S	✓	✓	✓		
Smith-Millman et al [64]	Kognito	Role-play/simulation		✓	✓		
Stone et al [65]	Youth suicide prevention: an introduction to gatekeeping	PowerPoint presentation, quiz/tests, resources, worksheet, audio files	✓	✓	✓		✓
Stover et al [66]	Pharm‐SAVES	Videos, reading material, resources	✓	✓	✓		
Sun et al [67]	Chinese life gatekeeper training program	Videos, role-play/simulation, contextual understanding, group discussion, Q and A session	✓	✓	✓		
Teo et al [68]	VA S.A.V.E.	Videos	✓		✓		
Teo et al [69]	VA S.A.V.E.	Videos, vignettes	✓	✓	✓		
Timmons-Mitchell et al [70]	Kognito At-Risk for Middle School Educators	Role-play/simulation	✓	✓	✓		
Wislocki et al [71]	Multiple (n=506)^f^	Videos	✓	✓	✓		

^a^We categorized topics in four categories: (1) Information about suicide (including information about suicide prevention, suicidal or self-injury behaviors, suicide myths, suicide prevalence and statistics, risk factors for suicide, protective factors against suicide, and signs of mental distress/suicidal ideation/warning signs), (2) Information about being a gatekeeper (including intervention skills and identification of at-risk individuals), (3) Information about resources and referrals, and (4) Information about general mental health (including mental fitness and self-care).

^b^We exclusively reported the topics explicitly mentioned in the article, but we acknowledge that the training programs might cover additional topics not mentioned in the article.

^c^MOOC: mass open online course.

^d^N/S: not specified.

^e^These systematic reviews and meta-analyses are included for thoroughness; however, data from the primary studies were not re-extracted in this table since most were already included individually, and the remaining did not meet our eligibility criteria.

^f^This scoping review included 506 training videos. For the sake of conciseness, we did not include the program names in this table.

### Data Analysis

We used an inductive content analysis approach [72,73] to identify the barriers and facilitators of web-based GTPs. Unlike the data extraction process, which aimed to summarize descriptive characteristics of the studies, the content analysis involved a separate, in-depth examination of the full texts of the included articles. EG and KW used Dedoose (version 9.0.107; Socio Cultural Research Consultants, LLC) to independently and inductively code full-text PDFs of the articles to reduce bias in interpretation. Coding began with line-by-line open coding, assigning initial code to relevant segments of text without predetermined categories. After 10% of the articles were coded, the two coders met to compare their coding frameworks and ensure consistency. Joined by KL, they then proceeded to independently code the remaining articles. This process allowed for a systematic and thorough examination of the data and minimized the risk of missing important nuances. Once all articles were coded, EG aggregated codes into higher-order headings, which were reviewed by the research team to develop broader categories through the process referred to as abstraction [72].

## Results

### Results of the Search

**Figure 1:**
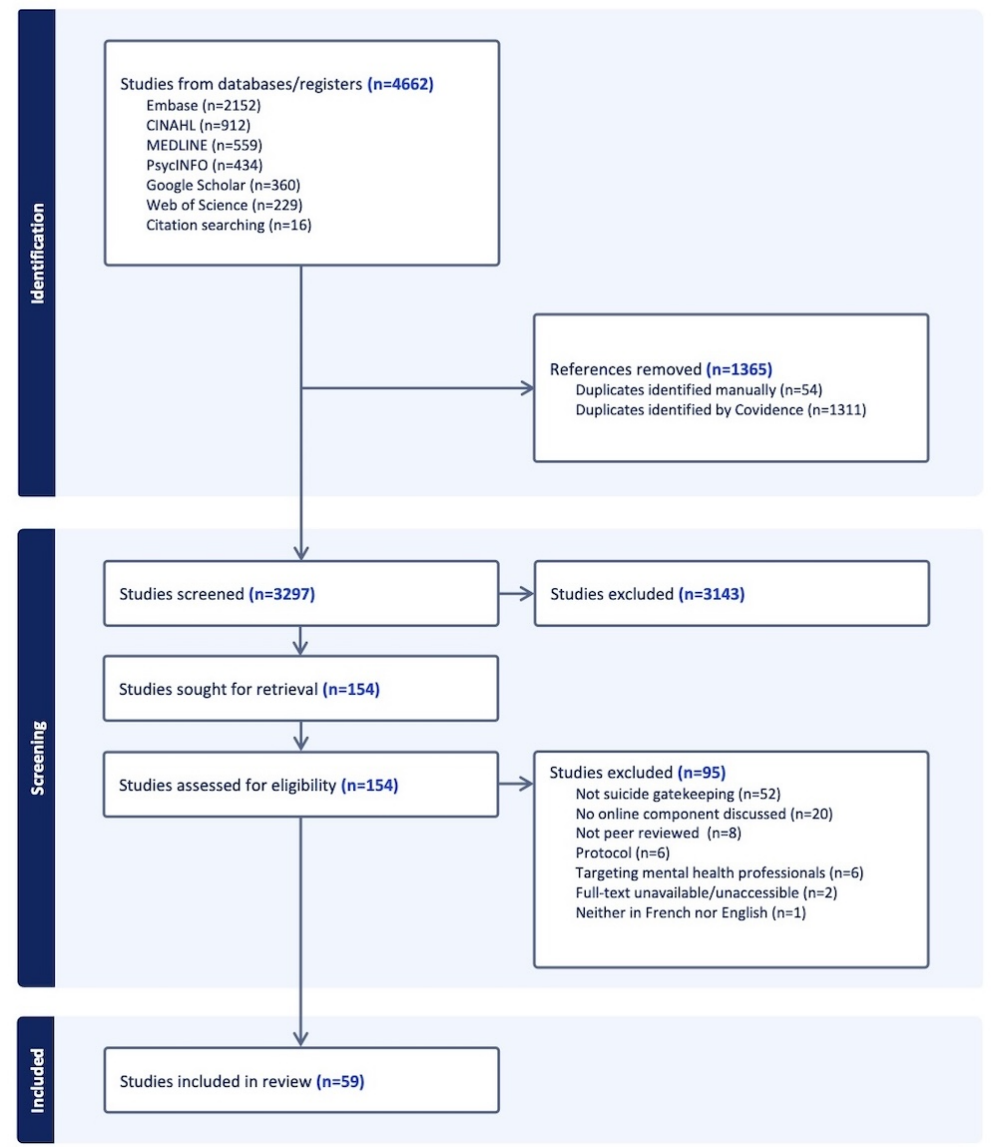
PRISMA flow diagram.

Our search yielded 4662 entries, from which 1365 duplicates were removed. After title and abstract screening, 3143 articles were excluded, and 95 articles were further removed after full-text screening (Figure 1). The final sample included 59 articles published between 2003 and 2025.

### Reports Included in the Synthesis

Among the 59 articles reviewed, 36 used quantitative methodologies, including 11 randomized controlled trials. Additionally, 10 articles discussed multiple training [7,8,10,24,39,42,60-62,71]. The most common training programs discussed were Kognito (n=7); S.A.V.E./SAVE (signs, ask, validate, and encourage/expedite) or pharm-SAVE (n=5); and question, persuade, refer (n=4; excluding counts from review articles). The program settings varied, with schools/universities (n=21), military or law enforcement environments (n=6), and health care/ clinical settings (n=4) being the most common. Training duration ranged widely, from 20 minutes [25] to 32 hours [50] or multiple weeks [26,63,65]. Most training programs were aimed at youth or students (n=13), school staff (n=9), health care providers (n=6), or parents/caregivers (n=3). A brief overview of the characteristics of the GTPs included in this review is presented in Table 1, while a complete version is provided in Multimedia Appendix 3.

### Design

We identified several critical design elements for web-based GTPs. Foremost, authors recommend that programs be grounded in theory and evidence-based practices [23,26,31,43,52,54,67,71]. For instance, Timmons-Mitchell et al [70] recommended evidence-based techniques such as motivational interviewing, and Afsharnejad et al [19] emphasized that mental health education principles, specifically the PERMA framework, should be used to enhance the efficacy of training. In addition, it was recommended that programs align with existing available suicide prevention initiatives to leverage and integrate resources [27,53,58]. This design specificity underlines web-based GTPs as a connector between community members and existing prevention efforts rather than being a stand-alone solution [22,43,54]. Indeed, it was emphasized by several authors that web-based GTPs should complement and be purposefully integrated rather than aspire to replace other suicide prevention initiatives [22,30,40,54,56]. To ensure usefulness and promote uptake, several programs integrated a co-design approach, involving interdisciplinary experts (including website developers), stakeholders, and potential end users participating in the design and implementation processes to better tailor the training programs [19,22,26,28,34,37,50,66-68,71]. In addition, the need for adequate financial support to sustain, maintain, and improve the programs to ensure the integration of new knowledge and best practices was mentioned [8,21].

Accessibility was also an essential design element. Authors highlighted that technical issues, such as glitches in program software, can hinder accessibility by disrupting the flow of the training [10,34,41,61]. Despite assumptions of widespread internet access, real-world barriers such as low bandwidth, limited availability of internet or technologies (eg, computers, smartphones), and insufficient media skills persist, potentially limiting users’ access [10,21,22,30,35,61]. Authors suggested that the training platforms should be more accessible by reducing the bandwidth requirements and ensuring 24/7 availability [8,53]. They also emphasized simplifying navigation [54] through avoiding registration [52], the use of specific software (eg, Adobe), or requiring specific web browsers [10]. Further, the variability of individual trainee competencies and preferences in terms of technology use necessitates that training platforms be user-friendly and ideally use technologies familiar to learners for a better user experience [22,48,52,57,70]. Brown et al [22] recommended the adaptability of training programs across multiple technological devices (eg, computer, smartphones, and tablets) and suggested incorporating diverse web-based supports (mobile app, Facebook group, messenger feature, etc). Accessibility was also contingent on delivering content through diverse formats, including audio, video, and text, and ensuring these vehicles could be adjusted to suit individual learning speeds and accommodate trainees with disabilities and learning or attention deficits. Practically, this means adding audio components to written material [8] with a sufficiently large font [10], being able to control the speed of audio and video content [30], and adding embedded text explanations and captions to videos [66]. Multiple studies [10,54,61] suggested adding simple, nongeneric visual components to text as well as color-coding sections to create a visual learning structure, avoiding long sentences to describe concepts and prioritizing bullet points or synthesized information. Authors also highlighted the importance of accessibility and program duration, noting that web-based training programs should be as brief as possible while still meeting learning objectives [10,20,25,34,37,41,44,46,54,57,69]. For example, trainees in the study by Carpenter et al [25] preferred the theoretical part of the training to last 30 minutes or less, and trainees in the study by Reifegerste et al [54] rated videos averaging 7-8 minutes to be too lengthy. Still, Liu et al [42] recommend that training sessions last more than 2 hours to induce a significant change in trainees’ attitudes and behaviors. Last, Carpenter et al [25] and Wislocki et al [71] also emphasized the importance of cost for the training programs, and making them financially feasible for a wide range of trainees.

Customizability was another critical element wherein the program design needed to be adaptable to meet trainees’ unique needs, personal characteristics, and professional backgrounds [8,22,26,27,31,35,41,43,53,54,57,64,71]. This approach comprised providing personalized feedback tailored to each trainee’s progress [10,21,57] and developing customized learning experiences for trainees based on their prior gatekeeping and suicide prevention experience and knowledge [8,22,32,37,54]. This included adding complementary modules, supplemental material, and additional content or offering flexible options for practice sessions [10,47]. Moreover, the customization of web-based training should encompass a variety of gatekeeping examples, scenarios, and practice exercises, thereby facilitating the alignment of content with diverse trainee preferences [10,25,37,41,66,70]. Furthermore, to facilitate customization that accommodates trainees’ variable schedules and pacing, authors recommend enhancing program flexibility through multiple learning modes and having the option to segment learning sessions [8,10,31,35,48,70].

Another design consideration for web-based training was standardization. Emphasized was the importance of implementing mechanisms to ensure consistency in the delivery of training content [31]. In web-based synchronous programs led by human instructors, standardization largely depends on the instructor’s expertise and familiarity with the material and the target audience, which was not specified in the reviewed articles. While instructors can adapt to trainees’ needs and pace, maintaining consistency remains crucial, especially given potential fatigue, which can affect delivery. In asynchronous programs, standardization was built into the training itself. To support this, authors have recommended incorporating elements such as standardized scenarios, automated feedback systems, and prerecorded videos to ensure uniformity across sessions [21,62]. These standardization mechanisms promote consistency and create a sense of psychological safety for trainees, as they reduce the risk of judgment from instructors or peers [21,62,70]. Bartgis et al [21] have recommended standardization in delivery mechanisms, including web-based role-play modalities using avatars, to ensure consistency in content delivery, regardless of trainees’ personal characteristics.

Another design consideration was privacy [10,22,28,42,46]. Reifegerste et al [54] noted that web-based formats may offer a greater sense of anonymity compared with in-person settings. While complete anonymity cannot be guaranteed in web-based training programs, Lancaster et al [9], Caughlan et al [26], and Reifegerste et al [54] argue that it remains an important feature, as it may reduce trainees’ anxiety, thereby encouraging more open engagement with sensitive topics. Some authors raised concerns about privacy risks associated with using third-party internet platforms. For example, Brown et al [22] noted that while using a private Facebook group can foster support and connections among trainees, it may also pose risks to participants’ privacy and confidentiality. Further, confidentiality concerns extended beyond the training itself to include program evaluation. Thus, it is essential that all data collected during the training, including during role-playing exercises, is stored on a secure server [21].

The final key element to consider in the design phase was sustainability. Specifically, maintaining and updating technological aspects were significant challenges for the use and longevity of web-based GTPs [8]. Brown et al [22] suggested that program designers allocate resources to ensure the maintenance and updates of the training in the initial development plan. For example, they specified that while web-based forums can be found valuable among trainees, they require substantial effort in terms of moderation and maintenance, which can be resource-intensive. Ghoncheh et al [15] emphasized the importance of developing a sustainability plan that minimizes maintenance and cost while preserving the integrity of the program.

### Content

A consistent content recommendation was the use of clear and concise terminology and vocabulary familiar to trainees [10,51,54,69]. In programs designed for nonprofessionals, avoiding technical terminologies or clinical jargon, which may act as a barrier, was a consistent recommendation [10,37,54]. Authors recommended balancing testimonials (“emotional content”) and practical information (“informative content”) to better engage trainees and support the destigmatization of suicide prevention [10,54]. In addition, the importance of activities that help trainees feel comfortable using the word “suicide” [25] and support them in discussing suicidality in a nonstigmatizing manner was emphasized [19,26,27,31,57]. At the same time, caution was advised against including graphic details of suicide death [57] or downplaying the gravity of the topic [47]. Authors of the included studies recommended providing a manageable amount of clear, straightforward, and easy-to-follow information to avoid overwhelming trainees [10,69] while still offering sufficiently rich content [47,54].

In terms of didactic topics to cover, trainees endorsed four different topics: (1) Information about suicide including suicide definition, epidemiology, statistics and prevalence [10,25,27,31,43,52,66], and suicide myths and beliefs [10,25,32,52]; contextual factors connected with suicide [26,30], content about at-risk subgroups [27,30,35,52,54], legal requirements or policies [10,25], and broader community concerns regarding suicide prevention [22,58]; (2) Information about how to be a gatekeeper including how to identify at-risk individuals [22] and the differentiation between warning signs and risk factors according to various settings [10,27,30,31,35,43,52,54,58,69,71], clear and memorable steps to follow for gatekeeping interventions [10,25,51,52,66], ways to initiate conversations about suicide [25,26,35,51,52,58,69], what to say and topics to avoid when talking with suicidal individuals [41,47,54,69], safety planning and problem-solving [35,41,71], including with dealing with nonreceptive individuals [50], and follow-up strategies postintervention and postvention care [22,41,66,71]; (3) Information about the broad range of existing services including referral guidance [10,22,25,27,30,35,42,43,45,50,52,54,69,71] according to existing local resources [10,21,25,26,66]; and (4) Information about general mental health for gatekeepers [42,54] including mental fitness [19] or mindfulness [41], the challenges of being a [22,25,30,31] and gatekeepers’ well-being and self-care strategies [22,35,41,57-60]. Importantly, these topics should be adapted to trainees’ backgrounds and accompanied by relevant examples to which trainees could relate [10,25,41,42,45,51,66] as well as the specific context of the intervention [10,22,30,43,47]. The importance of ensuring that the content was culturally relevant to the intended audience was described as a factor bolstering the effectiveness and the inclusivity of training programs [7,21,22,26,27,30,38,42,53,63,67]. Notably, 4 studies underscored the limited diversity in training, pointing to a lack of content and examples specifically addressing the needs of LGBTQ+ populations and women [28,41,54,71]. In contrast, topics perceived as less relevant by trainees included procedural guidance on reporting suicide cases, professional assessment practices that were not directly applicable to their roles or lived experiences, and research data from unrelated contexts [22,30].

In addition to the topics above, several core skills and competencies to be acquired by trainees were highlighted. These include the ability to establish a strong rapport and build a trusting relationship with individuals experiencing suicidal ideation [22,30]. Brown et al [22] and Hawley et al [31] emphasized the inclusion of interpersonal “soft skills”—including active listening, compassion, patience, and nonjudgmental attitudes—as foundational elements of effective training. Similarly, Bartgis et al [21] underscored the importance of motivational interviewing skills, including the use of open-ended questions, providing affirmation, reflective listening, and summarizing. The development of advocacy skills was also identified as a particularly important skill when supporting individuals living in marginalizing conditions who face significant structural barriers to services [22].

### Pedagogy

Several pedagogical elements were identified as critical for the effective delivery of web-based gatekeeper training. First, the inclusion of interactive learning activities—including role-plays, hands-on activities, practice, and scenario-based exercises—was consistently emphasized as essential to skills development and enhancing learning outcomes [8,10,24,25,36,41,47,53,57,63,64]. Trainees highly valued these activities [10,22,23,25,26,30,37,41,45,51,62,69]. For example, participants in 4 studies [10,41,47,51] emphasized the importance of having more time allocated for practice during the web-based training through hands-on exercises and interactive learning opportunities to enhance motivation and information retention. Ross et al [59] also suggested having small group sizes to increase participation and knowledge gains in the case of synchronous training. Participants in 5 studies [10,25,47,57,66] endorsed role-plays that were concrete, realistic, relatable, and applicable to their contexts. Although some authors noted challenges for implementing role-plays via web [15,30,51], Seabury [62] and Liu et al [42] encouraged leveraging the use of innovative technologies. Examples of such technologies include the use of avatars [21,23,29,55,60,64], virtual reality [60], interactive videos or video demonstrations [41,51,66], live videoconferencing role-play practice sessions [10], or mathematical behavioral models and algorithms to create realistic simulations where trainees can practice gatekeeper skills [70]. Knowledge checks, such as tests and quizzes, were identified as another vital component of interactive learning [8,10,34,41], providing trainees with immediate feedback on their understanding of the material covered [61]. However, some authors cautioned that web-based training might lack the interactive and adaptable learning environments synonymous with traditional in-person training programs where instructors could respond to trainees in real-time [62,71]. Offering clarifications and real-time feedback dispersed throughout the training, including via knowledge checks, could address this limitation [8,10,41,62].

A second key pedagogical element was the autonomy for trainees regarding the pace and style of learning [8,9,19,21,34,35,62]. Inversely, several authors cautioned against over-reliance on trainees’ intrinsic motivation [9,20], which can limit engagement [10,19] and even increase attrition [65]. Thus, authors emphasized that it is crucial to implement ongoing guidance, learner incentives (eg, raffle for a gift certificate [44]), and motivational strategies (eg, email reminders to finish the training [37]), given that intrinsic motivation may not be sufficient to complete the training [19,41,63]. Such mechanisms could take the form of a time limit for training completion [20] or reminders to encourage trainees to complete the training [37,41].

Building a community of practice emerged as another key recommendation. Unlike face-to-face training, web-based training programs often limit direct interactions with trainers as well as with other trainees. This lack of human interactions was mentioned as hindering skills practice and development [19,21]. Afsharnejad et al [19] highlighted the importance of fostering emotional connection with trainees. Establishing a community of practice, as suggested by other authors, could support connectivity by enabling trainees to access expert insights and feedback [8,41,53,57]. Some also highlighted that such communities of practice could provide trainees with opportunities to be paired with gatekeeping buddies for peer support [22], offering continuing networking and debriefing opportunities [22,38], offering support [59], and contributing to shared learning [51]. Examples of implemented approaches include messaging platforms [57], moderated forums with discussion boards and threads [8,65], chatrooms [28], and digital coaches providing direct and personalized feedback [21].

Finally, reinforcement strategies were recommended to consolidate skills and knowledge acquired during web-based training. This could include incorporating multiple repetitions of the learning material throughout the training [8]. While repetition was mentioned to maximize learning, participants in the studies by Kimbrel et al [37] and Kreuze et al [10] indicated that repetition was irritating, distracting, and even useless. Other recommendations included follow-up training sessions spaced in time to reinforce and sustain knowledge [31,42,51], training refresher sessions [15,21,22,37,42,51,53,56,69]—although Kingi-Ulu’ave et al [38] concluded that passive boosters were not impacting knowledge retention and trainees’ self-efficacy—and complementary material like digital workbooks to write notes, take-home training summaries, and complementary resources [10,22,34,35,41,51].

Overall, Kreuze et al [10] recommended having a variety of teaching and evaluating strategies to address diverse learning styles and needs, promote critical thinking, and incorporate learning across the cognitive, affective, and psychomotor domains (eg, through role-plays).

An integrated list of dos and don’ts, organized from the results, is provided as a one-page checklist in Multimedia Appendix 4 [8-10,14,15,19-54,56-71,74].

## Discussion

### Principal Findings

This narrative review systematically synthesized findings drawn from published literature focused on web-based suicide prevention GTPs to make recommendations for key design, content, and delivery. While there is increasing enthusiasm for web-based education broadly [75,76], including in the field of suicide prevention [9,77], there is a lack of clear guidance on best practices for the development and implementation of these programs [14]. The findings of this review contribute to addressing this knowledge gap by detailing critical elements across 3 key areas: design, content, and pedagogy. These insights offer a conceptual foundation for future research and offer practical guidance for the development and implementation of effective web-based gatekeeper training initiatives.

A central finding of this review is the necessity of grounding program design in evidence and theory, both in terms of the GTP content and its pedagogy. Integrating evidence-based practices in its content not only enhances the credibility of gatekeeper training but is also essential for ensuring its effectiveness. Yet, this is not without challenges. Evidence-based suicide prevention initiatives remain in early stages of translation [78], and a key critique of GTPs is the frequent lack of rigorous evaluation that can demonstrate their effectiveness [79]. In terms of pedagogy, most programs to date draw from social cognitive theory [80] and the theory of planned behavior [81], which highlight psychosocial determinants of behavior change and draw attention to contextual factors that influence how new behaviors are learned and sustained. However, these theories focus on individual behaviors and may fall short of accounting for the specific affordances and constraints of digital learning environments [82]. The inclusion of instructional design theories tailored to web-based modalities—such as the community of inquiry framework for web-based learning [83] and the cognitive theory of multimedia learning [84]—could significantly strengthen the pedagogical foundation of these programs, taking full advantage of web-based platforms.

Moreover, research and existing guidelines suggest that effective gatekeeper training should be designed and situated within a broader, multilevel suicide prevention strategy [1]. This aligns with recommendations from the WHO [1] and multiple reviews of national suicide prevention strategies [85-87], which recognize the potential of GTPs and their limitations as a stand-alone intervention (Zalsman et al [79]). For example, GTPs are not meant to replace suicide specific interventions delivered by trained mental health professionals. In addition, the effectiveness of GTPs may be limited in environments where suicide is highly stigmatized [88]. Indeed, stigma has consistently been identified as a key factor contributing to gatekeepers’ reluctance to intervene [4]. It can also play a complicit role in silencing individuals with suicidal thoughts [12,89]. The threat of stigma is especially relevant for marginalized populations, including LGBTQ+ and racialized populations, who already experience significant barriers to safe and appropriate mental health care [90,91]. Moreover, while these programs can help connect individuals experiencing suicidal thoughts to appropriate support, there is little evidence on how they foster hope or promote a meaningful life [12,92]. In addition to building core gatekeeper competencies—identifying warning signs, talking about suicide, referring—future GTPs should incorporate upstream approaches that actively promote mental health well-being and reduce stigma.

To better tailor GTPs to current needs, several articles recommended co-design approaches in the development of web-based GTPs. Co-design approaches have been described in the health literature as an effective approach to enhance program relevance and user engagement by involving end users in the development process [93,94]. However, as identified by Qasim et al [95], important knowledge gaps remain on how to best mobilize co-design approaches in maximizing outcomes. A useful starting point to optimize the inclusion of end users’ insights and needs in the co-design process could be to draw from participatory and community-based research principles [96]. Despite the espoused widespread use of participatory and community-based methods with vulnerable populations, there is still very little consensus on best practices [97,98]. Nevertheless, emerging key components such as fostering collaborative spaces [99,100], building capacities [98,101], and the balancing of power [102-104] could inform co-design practices of future GTPs.

In terms of content and pedagogy, this review reinforces the need for flexibility and adaptability to accommodate the varied needs and learning styles of trainees, aligning with broader findings on adult education and training programs [105]. Customizability—including tailoring scenarios, examples, and resources to specific user groups—has been shown to enhance engagement and practical application in education [106]. Dreier et al [16] suggest that customization of the training should also include the “desired degree of confrontation”—that is, text, images, videos, testimonials, representation of suicidality—not only to improve the engagement and satisfaction of the learners, but also to provide a sense of agency regarding the possible emotional distress in reaction to sensitive content. Interactive elements like role-plays, simulations, and real-time feedback are frequently cited as best practices in the education literature and are particularly effective in improving skill retention and building confidence [107-109]. While the adaptability and flexibility of web-based training have been extensively acknowledged as a strength in logistical terms (eg, pace and access) and range of possible content [110], they have often been criticized for their lack of interpersonal interactions, a key component to enhancing learning outcomes [111]. Among the reviewed articles, incorporating ways to foster a sense of connection and community among trainees in a web-based format (eg, creating or mobilizing already existing peer support networks and collaborative learning opportunities) has been emphasized and is consistent with research on social learning theory [112].

As a possible alternative to actual interpersonal interaction through the building of peer networks for collaborative learning, some authors have suggested the use of avatars or models—whose machine learning algorithm was not specified in the current reviewed articles—that would provide in vivo feedback in response to the trainees’ interactions. Such use of recent technologies provides an interesting avenue for interactive learning when actual human interaction is not possible. Although technologies based on artificial intelligence (AI) have been adopted in education—whether through students’ initiative or within the actual teaching curriculum—the absence of guidelines and best practices limits their adoption. In their systematic review of the use of AI in higher education, Ouyang et al [113] discussed the potential of AI technologies for improving the learner’s experiences in terms of engagement and providing accurate predictions (ie, real-time feedback). Yet, they further claim that the mere use of AI technologies does not necessarily lead to positive educational outcomes, emphasizing the importance of integrative frameworks or theories to support and enhance their ethical use. Another key ethical consideration in the use of AI technologies and open-source machine learning models is related to issues of confidentiality and privacy [114,115]. Such precautions become even more important in the context of vulnerable and sensitive topics such as suicide. Concerns about the privacy of information may also limit trainees in their willingness to participate in real-time AI-based role-play or feedback sessions. To mitigate risks and barriers to engagement, GTPs should explicitly disclose their use of such technologies, ensure secure data storage (eg, encrypted servers and restricted access), and implement clear protocols for handling data from role-play or training recordings [116].

Key barriers to the successful implementation of web-based GTPs for suicide prevention were identified in this review, many of which are inherent to web-based education in general. In addition to the lack of interpersonal and customizable human interactions, accessibility—while a strength—remains a significant challenge in terms of limited internet bandwidth, lack of access to the necessary technology, and varying levels of digital literacy impeding program reach and effectiveness. While the digital divide is narrowing globally [117], it continues to be a barrier for some populations due to persisting structural inequities flowing to and from the social determinants of health, including income, geographic location (ie, underdeveloped areas), and educational disparities [117,118]. Further, technical difficulties, including platform glitches and inefficiencies, not only hinder user experience and engagement but also the retention of learnings [119].

Sustainability is a critical challenge for web-based suicide GTPs. Without dedicated long-term funding, government, or commercial support, updates to keep content relevant and evidence-based are not possible. In fact, some of the interventions reviewed in this study could not be accessed at the time of writing, suggesting potential challenges to sustainability or ongoing availability. Ensuring long-term success requires more than just the initial development and implementation of an intervention. Sustainability should be taken into consideration in the initial steps of program development, given that it demands ongoing engagement, adaptation to evolving needs, and seamless integration into broader suicide prevention frameworks. The successful implementation of GTPs and their sustainability could benefit from network interventions at the public health level that focus attention on forging and solidifying intersectoral partnerships towards a more distributive model of responsibilities and accountability for suicide prevention [120].

### Future Directions for Research on Web-Based GTPs

Multiple calls have been made for more robust research into the evaluation of GTPs [4,79,121], and while we agree with this need, we also emphasize that understanding the most effective ways to deliver GTPs in web-based formats is important. Future studies should explore questions such as: “What are the most effective web-based approaches for training gatekeepers?” “What specific features of web-based training (eg, interactive modules, live simulations, peer discussions) can enhance skill acquisition and retention?” “How does web-based GTPs impact participants’ ability to recognize and respond to suicidal crises compared to in-person training?” Additionally, process evaluations and user feedback are essential to assess the quality of training delivery, user engagement, and practical application of skills learned via the web. Such evaluations can help to better understand implementation barriers and facilitators to improve the overall design of web-based GTPs. Another aspect to consider in the evaluation and design of GTPs is the impact and best delivery options of refreshers and booster sessions. While there is growing attention in tailoring GTPs to the needs of at-risk populations (eg, indigenous youth, migrant youth, gamers, and veteran as seen in this review), future research should also explore how web-based formats can better reach underrepresented and marginalized populations—such as LGBTQ+, Black, Indigenous and Racialized communities, or geographically isolated communities—more effectively and assess the training’s scalability and sustainability. Mixed methods studies and comparative trials are critical to advancing knowledge about how web-based GTPs can contribute to comprehensive suicide prevention strategies.

### Limitations

This review is limited by its focus on peer-reviewed articles published in English and French, potentially excluding relevant studies in other languages or formats (gray literature), which may introduce selection bias. The heterogeneity of included studies, such as variations in design, target populations, and program settings, made it difficult to draw generalizable conclusions. Many studies lacked rigorous evaluation methods, limiting our understanding of the impacts of the GTPs included. Additionally, insufficient reporting on user feedback, contextual factors (such as cultural and technological differences), and the implementation process restricts the applicability of findings across diverse settings. Notably, none of the studies reviewed specifically addressed our research question, which is, “what works” and “does not work” in web-based GTP. This gap underscores the need for further research that directly explores effective strategies to deliver web-based suicide prevention training to provide definitive guidance and improve training outcomes.

### Conclusions

Web-based GTPs hold significant promise for suicide prevention, yet much of the existing research has focused on determining whether these programs are effective [7], with evidence inconclusive as stand-alone interventions [88,122,123]. To advance the field, it is crucial to enhance evaluation efforts [123] while simultaneously exploring what works best in web-based formats for building gatekeeper skills. While there is a call for randomized controlled trials to build evidence of suicide prevention interventions such as GTPs to prove their efficacy, the next logical step in the evaluation and development of effective web-based GTPs should focus attention on the process of their implementation, including acceptability and feasibility. This review highlights a wide range of key considerations regarding web-based pedagogy that require closer attention to determine which might represent best practices to implement in the development of future GTPs. This involves a commitment to continuous improvement by leveraging technological advancements to enhance accessibility, engagement, and adaptability for diverse populations. By aligning program design with the latest innovations, including emerging tools such as AI [124,125], and rigorously evaluating their impact, web-based GTPs can evolve into a vital component of comprehensive suicide prevention strategies. Ultimately, these efforts will not only improve the quality and reach of interventions but also have the power to save lives.

## Appendix

Multimedia Appendix 1 PRISMA checklist.

Multimedia Appendix 2 Search strategy.

Multimedia Appendix 3 Complete table of characteristics of the online gatekeeper training programs included in the systematic narrative review.

Multimedia Appendix 4 Online gatekeeper training programs (GTP): Do’s and Don’ts checklist.

## Abbreviations

AI: artificial intelligence
GTP: gatekeeper training program
LGBTQ+: lesbian, gay, bisexual, trans, queer, and other sexual and gender minorities
PRISMA: Preferred Reporting Items for Systematic Reviews and Meta-Analyses
S.A.V.E./SAVE: signs, ask, validate, encourage/expedite
WHO: World Health Organization

## Data Availability

Data sharing is not applicable to this article as no datasets were generated or analyzed during this study.
