# Supplementary material for: Key Components and Barriers in Web-Based Suicide Prevention Gatekeeper Training: Systematic Narrative Review
Source: J Med Internet Res. 2026 Feb 5;28:e81572. doi: 10.2196/81572 (PMC12921433; doi:10.2196/81572)
Supplement: Multimedia Appendix 2 [file jmir_v28i1e81572_app2.docx]

**Concepts**

- Suicide
- Gatekeeper
- Online

**Databases**

- **Psychosocial**: PsycINFO
- **Biomedical :** Medline, EMBASE, CINAHL
- **Social sciences :** Web of science
- **Other:** google scholar

**Inclusion criteria**

- Published & peer-reviewed articles (all types)
- Language: English or French
- Date: 2000 – current
- Involving a suicide intervention that was provided online

**Exclusion criteria**

- Studies involving general mental health programs **not specific to suicide** (e.g., Mental Health First Aid Training)
- Training provided to mental health professionals
- Thesis, dissertation, *mémoires,* and grey literature
- Protocols or RCT registrations

| **Concepts** | **Concept 1**  Suicide prevention | **Concept 2**  Gatekeeper (training/program/initiatives) | | **Concept 3**  Online | |  |
| --- | --- | --- | --- | --- | --- | --- |
| **Keywords** | - Suicid* (exp) - Self killing or self-killing | - First assist*/aid/help/contact - gatekeep* or gate keep* - peer assist*/aid/help/contact* - peer educator* - peer group* - sentinel* - risk assessment training* - risk management training* | | - Android - app or apps or application* - Cellphone*, cell phone* - Chat-room* or chat room* - Computer* - Cyber* - Digital - E-intervention - Electronic intervention, electronic-intervention - Electronic mail* - Email*, e-mail* - Electronic tablet*, e-tablet* - Instant messag* - Internet - Internet-based - Ipad, i-pad - Iphone, i-phone - Messag* service* - Mobile* - Online, on-line - Portable Software App* - Portable electronic app* - Smartphone*, smart phone* - Sms - Social media* - Software - Technolog* - Telecomm* - Text messag*, text-messag* - Texting - Video-based, video based - Virtual - Web* - Wireless | |  |
| **Descriptors (MeSH)** | - [Suicide](https://www.ncbi.nlm.nih.gov/mesh/68013405) (exp) | - [First aid](https://www.ncbi.nlm.nih.gov/mesh/68005392) - [Peer group](https://www.ncbi.nlm.nih.gov/mesh/68010379) - [Psychosocial intervention](https://www.ncbi.nlm.nih.gov/mesh/?term=psychosocial+intervention) - [Social support](https://www.ncbi.nlm.nih.gov/mesh/68012944) (exp) | | - [Microcomputers](https://www.ncbi.nlm.nih.gov/mesh/68008838) (exp) - [Computer-Assisted Instruction](https://www.ncbi.nlm.nih.gov/mesh/68003194) - [Internet](https://www.ncbi.nlm.nih.gov/mesh/68020407) (exp) - [Online social networking](https://www.ncbi.nlm.nih.gov/mesh/2027952) - [Software](https://www.ncbi.nlm.nih.gov/mesh/68064878) (exp) - [Technology](https://www.ncbi.nlm.nih.gov/mesh/68013672) (exp) - [Telecommunications](https://www.ncbi.nlm.nih.gov/mesh/68013685) (exp) - [Virtual reality](https://www.ncbi.nlm.nih.gov/mesh/2023512) (exp) | |  |
| **Medline equation** | ((suicid* or (self ADJ2 killing)).ab,hw,kw,kf,ti. OR suicide (exp)) AND ((gatekeep* OR "gate keep*" OR sentinel* OR ((first or peer) ADJ2 (assist* or aid or help* or contact*)) OR (peer ADJ2 (group* OR educator*)) OR "risk assessment training*" OR "risk management training*").ab,hw,kf,kw,ti. OR first aid OR peer group OR social support (exp) OR psychosocial intervention) AND ((android OR app OR apps OR application* OR cellphone OR "cell phone" OR "chat-room*" OR "chat room*" OR computer* OR cyber* OR digital OR "e-intervention" OR "electronic intervention*" OR "electronic-intervention*" OR "electronic mail*" OR email* OR "e-mail*" OR "electronic tablet*" OR "e-tablet*" OR "instant messag*" OR internet OR "interned-based" OR ipad* OR "i-pad*" OR iphone* OR "i-phone*" OR "messag* service*" OR mobile* OR online OR "on-line" OR "portable software app*" OR "portable electronic app*" OR smartphone* OR "smart phone*" OR sms OR "social media*" OR software* OR technolog* OR telecomm* OR "text messag*" OR "text-messag*" OR texting OR "video-based" OR "video based" OR virtual OR web* OR wireless).ab,hw,kw,kf,ti. OR microcomputers (exp) OR Computer-Assisted Instruction OR internet (exp) OR online social networking OR software (exp) OR technology (exp) OR telecommunications (exp) OR virtual reality (exp)) | | | | | |
| **Descriptors**  **(EMTREE)** | - Suicidal behavior (exp) | | - First aid - Peer group - Psychosocial intervention - Social care (exp) | | - Computer simulation (exp) - Mass communication (exp) - online social network - Personal computer (exp) - Personal digital assistant - Software (exp) - Technology (exp) - Videorecording (exp) - Virtual reality system (exp) | |
| **EMBASE equation** | ((suicid* or (self ADJ2 killing)).ab,hw,kw,kf,ti. OR Suicidal behavior (exp)) AND ((gatekeep* OR "gate keep*" OR sentinel* OR ((first or peer) ADJ2 (assist* or aid or help* or contact*)) OR (peer ADJ2 (group* OR educator*)) OR "risk assessment training*" OR "risk management training*").ab,hw,kf,kw,ti OR First aid OR Peer group OR Psychosocial intervention OR Social care (exp)) AND ((android OR app OR apps OR application* OR cellphone OR "cell phone" OR "chat-room*" OR "chat room*" OR computer* OR cyber* OR digital OR "e-intervention" OR "electronic intervention*" OR "electronic-intervention*" OR "electronic mail*" OR email* OR "e-mail*" OR "electronic tablet*" OR "e-tablet*" OR "instant messag*" OR internet OR "interned-based" OR ipad* OR "i-pad*" OR iphone* OR "i-phone*" OR "messag* service*" OR mobile* OR online OR "on-line" OR "portable software app*" OR "portable electronic app*" OR smartphone* OR "smart phone*" OR sms OR "social media*" OR software* OR technolog* OR telecomm* OR "text messag*" OR "text-messag*" OR texting OR "video-based" OR "video based" OR virtual OR web* OR wireless).ab,hw,kw,kf,ti. OR Computer simulation (exp) OR Mass communication (exp) OR online social network OR personal computer (exp) OR personal digital assistant OR Software (exp) OR Technology (exp) OR Videorecording (exp) OR Virtual reality system (exp)) | | | | | |
| **SU**  **(PsycInfo)** | - Suicidal behavior (exp) - Suicide prevention - suicidality | | - Peers - Peer counseling - Peer relations - Psychological first aid - Social support (exp) | | - **Communications media** (exp) - **Digital interventions** - **Digital mental health resources** - **Human technology interaction** (exp) - **Information and Communication technology** (exp) - **Internet** (exp) - **Microcomputers** (exp) - **Mobile devices** (exp) - **Technology** | |
| **PsycInfo equation** | ((suicid* or (self ADJ2 killing)).ab,hw,ti,id. OR suicidal behavior (exp) OR suicide prevention OR suicidality) AND  ((gatekeep* OR "gate keep*" OR sentinel* OR ((first or peer) ADJ2 (assist* or aid or help* or contact*)) OR (peer ADJ2 (group* OR educator*)) OR "risk assessment training*" OR "risk management training*").ab,hw,ti,id. OR Peers OR peer counseling OR peer relations OR psychological first aid OR social support (exp)) AND ((android OR app OR apps OR application* OR cellphone OR "cell phone" OR "chat-room*" OR "chat room*" OR computer* OR cyber* OR digital OR "e-intervention" OR "electronic intervention*" OR "electronic-intervention*" OR "electronic mail*" OR email* OR "e-mail*" OR "electronic tablet*" OR "e-tablet*" OR "instant messag*" OR internet OR "interned-based" OR ipad* OR "i-pad*" OR iphone* OR "i-phone*" OR "messag* service*" OR mobile* OR online OR "on-line" OR "portable software app*" OR "portable electronic app*" OR smartphone* OR "smart phone*" OR sms OR "social media*" OR software* OR technolog* OR telecomm* OR "text messag*" OR "text-messag*" OR texting OR "video-based" OR "video based" OR virtual OR web* OR wireless).ab,hw,ti,id. OR communications media (exp) digital interventions OR digital mental health resources OR human technology interaction (exp) OR information and communication technology (exp) OR internet (exp) OR Microcomputers (exp) OR mobile devices (exp) OR technology) | | | | | |
| **Descriptors (CINAHL)** | - Suicide - Suicidal Ideation - Suicide Prevention - Suicide, Attempted | | - Peer assistance programs - Peer counseling - Peer group - Psychological first aid - Psychosocial intervention - Support, social (dev) - Support, psychosocial (dev) | | - Computer communication networks (dev) - Digital technology (dev) - Microcromputers (dev) - Online social networking - Software (dev) - Technology - Telecommunications (dev) - Videorecording (dev) - Virtual reality | |
| **CINAHL equation** | ((MH "Computer Communication Networks") OR (MH "Client-Server Application") OR (MH "Web Browsers") OR (MH "Electronic Bulletin Boards+") OR (MH "Usenet") OR (MH "Extranet") OR (MH "Internet+") OR (MH "Internet Access") OR (MH "Internet Connections") OR (MH "Internet-Based Intervention") OR (MH "Social Media+") OR (MH "Facebook") OR (MH "Twitter") OR (MH "World Wide Web+") OR (MH "Website Development") OR (MH "World Wide Web Applications") OR (MH "Web Search Engines") OR (MH "Internet of Things") OR (MH "Email") OR (MH "Internet Protocols") OR (MH "Digital Technology+") OR (MH "Microcomputers+") OR (MH "Computers, Portable+") OR (MH "Computers, Hand-Held+") OR (MH "Smartphone") OR (MH "IBM Compatible Microcomputers") OR (MH "Macintosh Microcomputers") OR (MH "Smart Glasses") OR (MH "Online Social Networking") OR (MH "Software") OR (MH "Communications Software+") OR (MH "Client-Server Application") OR (MH "Patient Portals") OR (MH "Computer Graphics") OR (MH "Hypermedia") OR (MH "Hypertext") OR (MH "Listserv") OR (MH "Mobile Applications") OR (MH "Operating Systems") OR (MH "Web Browsers") OR (MH "Technology") OR (MH "Telecommunications") OR (MH "Electronic Bulletin Boards") OR (MH "Email") OR (MH "Instant Messaging") OR (MH "Emoticons and Emojis") OR (MH "Interactive Voice Response Systems") OR (MH "Internet+") OR (MH "Internet Access") OR (MH "Internet-Based Intervention") OR (MH "Social Media+") OR (MH "Facebook") OR (MH "Twitter") OR (MH "Telecommuting") OR (MH "Teleconferencing") OR (MH "Telefacsimile") OR (MH "Cellular Phone+") OR (MH "Text Messaging") OR (MH "Smartphone") OR (MH "Voice Mail") OR (MH "Videoconferencing+") OR (MH "Webcasts+") OR (MH "Webinars") OR (MH "Wireless Communications") OR (MH "Videorecording+") OR (MH "Virtual Reality")) OR (AB ( (android OR app OR apps OR application* OR cellphone OR "cell phone" OR "chat-room*" OR "chat room*" OR computer* OR cyber* OR digital OR "e-intervention" OR "electronic intervention*" OR "electronic-intervention*" OR "electronic mail*" OR email* OR "e-mail*" OR "electronic tablet*" OR "e-tablet*" OR "instant messag*" OR internet OR "interned-based" OR ipad* OR "i-pad*" OR iphone* OR "i-phone*" OR "messag* service*" OR mobile* OR online OR "on-line" OR "portable software app*" OR "portable electronic app*" OR smartphone* OR "smart phone*" OR sms OR "social media*" OR software* OR technolog* OR telecomm* OR "text messag*" OR "text-messag*" OR texting OR "video-based" OR "video based" OR virtual OR web* OR wireless) ) OR TI ( (android OR app OR apps OR application* OR cellphone OR "cell phone" OR "chat-room*" OR "chat room*" OR computer* OR cyber* OR digital OR "e-intervention" OR "electronic intervention*" OR "electronic-intervention*" OR "electronic mail*" OR email* OR "e-mail*" OR "electronic tablet*" OR "e-tablet*" OR "instant messag*" OR internet OR "interned-based" OR ipad* OR "i-pad*" OR iphone* OR "i-phone*" OR "messag* service*" OR mobile* OR online OR "on-line" OR "portable software app*" OR "portable electronic app*" OR smartphone* OR "smart phone*" OR sms OR "social media*" OR software* OR technolog* OR telecomm* OR "text messag*" OR "text-messag*" OR texting OR "video-based" OR "video based" OR virtual OR web* OR wireless) ) OR SU ( (android OR app OR apps OR application* OR cellphone OR "cell phone" OR "chat-room*" OR "chat room*" OR computer* OR cyber* OR digital OR "e-intervention" OR "electronic intervention*" OR "electronic-intervention*" OR "electronic mail*" OR email* OR "e-mail*" OR "electronic tablet*" OR "e-tablet*" OR "instant messag*" OR internet OR "interned-based" OR ipad* OR "i-pad*" OR iphone* OR "i-phone*" OR "messag* service*" OR mobile* OR online OR "on-line" OR "portable software app*" OR "portable electronic app*" OR smartphone* OR "smart phone*" OR sms OR "social media*" OR software* OR technolog* OR telecomm* OR "text messag*" OR "text-messag*" OR texting OR "video-based" OR "video based" OR virtual OR web* OR wireless) )) | | | | | |
| **Web of science equation** | ((suicid* or (self NEAR/2 killing)).ti,ab,ts,ak,kp) AND ((gatekeep* OR "gate keep*" OR sentinel* OR ((first or peer) NEAR/2 (assist* or aid or help* or contact*)) OR (peer NEAR/2 (group* OR educator*)) OR "risk assessment training*" OR "risk management training*").ti,ab,ts,ak,kp AND ((android OR app OR apps OR application* OR cellphone OR "cell phone" OR "chat-room*" OR "chat room*" OR computer* OR cyber* OR digital OR "e-intervention" OR "electronic intervention*" OR "electronic-intervention*" OR "electronic mail*" OR email* OR "e-mail*" OR "electronic tablet*" OR "e-tablet*" OR "instant messag*" OR internet OR "interned-based" OR ipad* OR "i-pad*" OR iphone* OR "i-phone*" OR "messag* service*" OR mobile* OR online OR "on-line" OR "portable software app*" OR "portable electronic app*" OR smartphone* OR "smart phone*" OR sms OR "social media*" OR software* OR technolog* OR telecomm* OR "text messag*" OR "text-messag*" OR texting OR "video-based" OR "video based" OR virtual OR web* OR wireless). ti,ab,ts,ak,kp) | | | | | |
| **Google Scholar equation** | (suicid* or (self AROUND(2) killing)) AND (gatekeep* OR "gate keep*" OR sentinel* OR ((first or peer) AROUND (2) (assist* or aid or help* or contact*)) OR (peer AROUND(2) (group* OR educator*)) OR "risk assessment training*" OR "risk management training*") AND (android OR app OR apps OR application* OR cellphone OR "cell phone" OR "chat room*" OR computer* OR cyber* OR digital OR "e intervention" OR "electronic intervention*" OR "electronic mail*" OR email* OR "electronic tablet*" OR "instant messag*" OR internet OR "interned based" OR ipad* OR iphone* OR "messag* service*" OR mobile* OR online OR "portable software app*" OR "portable electronic app*" OR smartphone* OR "smart phone*" OR sms OR "social media*" OR software* OR technolog* OR telecomm* OR "text messag*" OR texting OR "video based" OR virtual OR web* OR wireless) | | | | | |
