# Supplementary material for: Key Components and Barriers in Web-Based Suicide Prevention Gatekeeper Training: Systematic Narrative Review
Source: J Med Internet Res. 2026 Feb 5;28:e81572. doi: 10.2196/81572 (PMC12921433; doi:10.2196/81572)
Supplement: Multimedia Appendix 3 [file jmir_v28i1e81572_app3.docx]

| **Author** | **Country** | **Study Design^1^** | **Training** | | | | | | | |
| --- | --- | --- | --- | --- | --- | --- | --- | --- | --- | --- |
|  |  |  | **Setting** | **Name** | **Online Format^2^** | **Duration (min)** | **Trainees** | **Target Population** | **Components of Training** | **Topics covered^3,4^** |
| Afsharnejad et al [1] | Australia | Quantitative; RCT | University | Talk-to-me MOOC | Synch  Asynch  N/S | 86.4 | University students | Young adults | Videos, quiz/tests | Information about suicide  Information about being a gatekeeper |
| Albritton et al [2] | USA | Quantitative; Pre/post evaluation | N/S | Be Present | Synch  Asynch  N/S | N/S | Youth | Youth | Videos, e-tool box, homework assignments, social media posting | Information about suicide |
| Bartgis and Albright [3] | USA | Quantitative; Pre/post evaluation | Schools and Universities | Kognito Gatekeeper Simulations (KGS) | Synch  Asynch  N/S | N/S | American Indian and Alaska Native students, teachers, and faculty staff | Youth and students | Role-play/simulation | Information about being a gatekeeper  Information about resources/referrals |
| Brown et al [4] | Australia | Qualitative; Thematic analysis | Indigenous communities | Indigenous Network Suicide Intervention Skills Training (INSIST) program | Synch  Asynch  N/S | N/S | Indigenous health workers or community members | Indigenous youth | N/S | N/S |
| Bryant et al [5] | USA | Quantitative;  Quasi-experimental pre/post evaluation | University | Kognito “At Risk Primary Care” | Synch  Asynch  N/S | 60 | Family nurse practitioner students | N/S | Role-play/simulation | Information about being a gatekeeper  Information about resources/referrals |
| Canady [6] | USA | Editorial | School | Signs Matter: Early Detection | Synch  Asynch  N/S | 120 | School staff^5^ | Students | Role-play/simulation quiz/tests, resources | Information about suicide |
|  |  |  |  | At-Risk for High School Educators |  | 60 |  |  |  | Information about suicide  Information about being a gatekeeper  Information about resources/referrals |
|  |  |  |  | At-Risk for Middle School Educators |  |  |  |  |  |  |
| Carpenter et al [7] | USA | Qualitative; Thematic analysis | Healthcare | Online Veteran Administration's (VA) suicide prevention gatekeeper training program (SAVE) | Synch  Asynch  N/S | 20 | Pharmacy staff members | Pharmacy clients/patients | Videos, role-play/simulation | Information about suicide  Information about being a gatekeeper |
| Caughlan et al [8] | USA | Mixed;  Multi-phased single-arm pre/post evaluation | N/S | Mind4Health | Synch  Asynch  N/S | 90 | Parents, coaches, counselors, and families | American Indian/ Alaska Native youth | Videos, readings, resources | Information about being a gatekeeper  Information about resources/referrals |
| Cohen et al [9] | Israel | Quantitative; Pre/post evalutation | Various community settings/work environment (schools, nursing homes…). | Israeli gatekeeper training | Synch  Asynch  N/S | 240 | Professional engaging with at-risk populations | Elderly, adolescents, and LGTBQ+ individuals | Role-play/simulation | Information about suicide  Information about resources/referrals |
| Colder Carras et al [10] | N/S | Qualitative; Case study | Military gaming community | Stack Up’s Overwatch Program (StOP) | Synch  Asynch  N/S | N/S | Soldiers, veterans or community members | Gamers, including military or veteran gamers | N/S | N/S |
| Coleman et al [11] | USA | Quantitative; Pre/post evaluation RCT | University | Kognito At Risk for College Students | Synch  Asynch  N/S | N/S | University students | University students | Role-play/simulation | Information about being a gatekeeper  Information about resources/referrals |
| Colucci et al [12] | Syria | Mixed; Pre/post evaluation | Low- and middle-income conflict-affected areas | Suicide First Aid Guidelines (SFAG) training | Synch  Asynch  N/S | 480 - 900 | Humanitarian workers | Migrant or refugee youth | Videos, quiz/tests, infographics, homework assignments, reflective journaling | Information about suicide  Information about being a gatekeeper  Information about resources/referrals |
| Ghoncheh et al [13] | Netherlands | Quantitative; Pre/post evaluation RCT | School and healthcare/ clinical setting | Mental Health Online (MHO) | Synch  Asynch  N/S | 32 - 80 | Healthcare providers and school staff^5^ | Youth | PowerPoint presentation, quiz/tests, discussion board | Information about suicide  Information about being a gatekeeper  Information about resources/referrals |
| Ghoncheh et al [14] | Netherlands | Qualitative; Descriptive review | N/S | Mental Health Online (MHO) | Synch  Asynch  N/S | 80 | Professionals working with adolescents including school staff^5^ | Youth | PowerPoint presentation, audio features, graphs, quiz/tests, reading material, discussion board | Information about suicide  Information about being a gatekeeper  Information about resources/referrals |
|  | UK |  |  | Children and Family Court Advisory and Support Service (Cafcass) program |  | N/S | Cafcass staff |  | N/S | N/S |
|  | USA |  |  | QPR Online Gatekeeper Training |  |  | N/S | Individual at-risk for suicide | PowerPoint presentation, role-play/simulation, quiz/tests, reading material, videos, audio features |  |
|  |  |  |  | Hollywood Homeless Youth Partnership (HHYP) program |  |  | Staff working with homeless youth | Youth | PowerPoint presentation, audio features, quiz/tests |  |
|  |  |  | Law enforcement | In the Line of Duty |  |  | Police officers | General population | Videos, audio features |  |
| Hawley et al [15] | USA | Quantitative; Pre/post evaluation | Various settings including educational, health, community | N/A | Synch  Asynch  N/S | 60 - 90 | University and school staff, students, health care workers, and community members | N/S | Didactic content, video and audio clips,and reflection questions | Information about suicide  Information about being a gatekeeper  Information about resources/referrals  Information about general mental health |
| Hill and McCray [16] | USA | Quantitative; Pre/post evaluation | University | The Texas AS + K? Suicide Gatekeeper Training Program | Synch  Asynch  N/S | 60 | University students | N/S | N/S | Information about suicide  Information about being a gatekeeper  Information about resources/referrals |
| Hill et al. [17] | USA | Quantitative; Pre/post evaluation RCT | University | ASK About Suicide to Save a Life (AS + K?) | Synch  Asynch  N/S | 60 | University students | N/S | Videos | Information about suicide  Information about being a gatekeeper |
| Hofmann et al [18] | Germany & Switzerland | Quantitative;  Pre/post quasi-experimental evaluation | Police department | COPS (Coping with Suicide) | Synch  Asynch  N/S | 60 | Police officers | Individuals at-risk for suicide | Videos, reading material, worksheets, quiz/tests | Information about suicide  Information about being a gatekeeper  Information about resources/referrals |
| Hofmann and Wagner [19] | Germany | Quantitative; Pre/post evalutation RCT | N/S | N/S | Synch  Asynch  N/S | N/S | Relatives of men experiencing suicidal ideation | Men at-risk for suicide | Videos, audio plays, manual | Information about suicide  Information about being a gatekeeper  Information about resources/referrals  Information about general mental health |
| Holmes et al [20] | Australie | Quantitative; Pre/post evaluation | N/S | Start | Synch  Asynch  N/S | 90 | Sunshine Coast, Queensland residents | N/S | N/S | N/S |
| Kawashima et al [21] | Japan | Quantitative; pre/post evaluation RCT | University | N/S | Synch  Asynch  N/S | 27 | University students | N/S | PowerPoint presentation, videos | Information about suicide  Information about being a gatekeeper  Information about resources/referrals |
| Kimbrel et al [22] | USA | Quantitative; Pre/post evaluation | Fire brigade | Safety Planning Intervention (SPI) | Synch  Asynch  N/S | 120 - 190 | Firefighter peer support specialists | Individuals at-risk for suicide and firefighters | PowerPoint presentation, videos, role-play/simulation, reading material, worksheets | Information about being a gatekeeper |
| Kingi-Ulu'av et al [23] | New Zealand | Quantitative; RCT | Community | LifeKeepers booster session | Synch  Asynch  N/S | N/S | Non-health professionals or community members | New Zealand communities | Readings | Information about suicide  Information about being a gatekeeper  Information about resources/referrals |
| Kingi-Ulu'av et al [24] | Australia and USA | Qualitative; Review of reviews | Community | Question Persuade and Respond (QPR) | Synch  Asynch  N/S | N/S | Salvation Army volunteers | Various including youth | N/S | N/S |
|  | Netherlands |  | N/S | Mental Health Online (MHO) | Synch  Asynch  N/S | 40 - 80 | Individuals working with adolescents | Adolescents |  |  |
|  | USA |  | University | I CARE | Synch  Asynch  N/S | 30 | School staff and students | N/S |  |  |
|  |  |  | School | Act on FACTS: Making Educators Partners (MEP) | Synch  Asynch  N/S | N/S | School staff | Students |  |  |
|  |  |  | School and university | Kognito Gatekeeper Simulations |  |  |  | N/S |  |  |
| Kreuze and Ruggiero [25] | USA | Qualitative; review | School | Kognito At-Risk for High School Educators | Synch  Asynch  N/S | N/S | School staff^5^ | Students | Videos, role-play/simulation | Information about being a gatekeeper  Information about resources/referrals |
|  |  |  | N/S | Question, Persuade, Refer (QPR) |  |  | N/S | N/S | Videos | Information about suicide  Information about being a gatekeeper  Information about resources/referrals |
|  |  |  | School | Making Educators Partners in Youth Suicide Prevention: ACT on FACTS |  |  | School staff^5^ | Youth | Videos, role-play/simulation, audio features |  |
| Kreuze et al [26] | USA | Mixed; Comparative evalution | School | Question, Persuade, Refer (QPR) | Synch  Asynch  N/S | 60 | School personnel | Youth | Videos, testimonials, narration, bulleted lists, mnemonics, pocket cards, role-play/simulation, self‐audit checklist | Information about suicide  Information about being a gatekeeper  Information about resources/referrals |
|  |  |  |  | Making Educators Partners in Youth Suicide Prevention (MEP) |  | 120 |  | Marginalized and bullied youth and those returning to school after a suicide attempt. | Videos lectures, expert content, conversations example, roles-play, testimonies, activities related to videos |  |
| Lamis et al [27] | USA | Quantitative; Pre/post evaluation | School | Making Educators Partners in Youth Suicide Prevention: ACT on FACTS | Synch  Asynch  N/S | 120 | School staff^5^ | Youth | Lecture, question and answers, virtual vignettes/interactive activities | Information about suicide  Information about being a gatekeeper  Information about resources/referrals |
| Lancaster et al [28] | Australia and USA | Quantitative; Pre/post evaluation RCT | N/S | Web-based Question, Persuade, and Refer (QPR) | Synch  Asynch  N/S | N/S | Australia Salvation Army volunteers and general population | Individuals at-risk for suicide | Videos, text, pictures, audio features | N/S |
| Lee-Tauler et al [29] | USA | Mixed; Descriptive pilot program evaluation | Military | Chaplains-CARE Online program | Synch  Asynch  N/S | 180 – 360+ | Military chaplains | Military service members | Didactic lectures, videos, reading material, quiz/tests, interactive activities | Information about suicide  Information about being a gatekeeper  Information about general mental health |
| Liu et al [30]^6^ | Germany, Japan, Netherlands, USA | Quantitative; Systematic review & meta-analysis of RCTs | Various | Various | Synch  Asynch  N/S | Various | Various | Various | Various | Various |
| MacDonald Hart et al [31] | UK, France and Belgium | Quantitative; Pre/post evaluation | Humanitarian | Suicide Intervention First Aid (SIFA) | Synch  Asynch  N/S | 540 | People providing aid to asylum-seeking and refugees | Asylum seeking and refugees | Didactic lectures, interactive, discussions, role-play/simulation exercise, skills practice | Information about suicide  Information about being a gatekeeper  Information about resources/referrals |
| Manning and Van Deusen [32] | USA | Program description | University | Western Michigan University (WMU) Suicide Prevention Program (SPP) Online Course | Synch  Asynch  N/S | 60 - 120 | School staff^5^ | University students | Videos, photographs and graphics | Information about suicide  Information about being a gatekeeper  Information about resources/referrals |
| Marley et al [33] | USA | Quantitative; Cross-sectional | N/S | Pharm-SAVES training | Synch  Asynch  N/S | 30 | Pharmacists and pharmacy staff | General population | N/S | Information about being a gatekeeper  Information about resources/referrals |
| McKay et al [34] | Australia | Quantitative; Pre/post evaluation | N/S | Living Works Start | Synch  Asynch  N/S | 90 | Parents and caregivers | Youth and young adults | Videos, reading material | Information about suicide  Information about being a gatekeeper  Information about resources/referrals |
| Mirick [35] | USA | Mixed; post evaluation | School | SOS Signs of Suicide for School Staff | Synch  Asynch  N/S | 60 | K-12 school staff | Youth, students | Role-play/simulation  with both child and adolescent | Information about suicide  Information about being a gatekeeper  Information about resources/referrals |
| Mishkind et al [36] | USA | Mixed;  Pre/post evaluation | Workplace | VitalCog: Suicide Prevention in the Workplace (formerly known as Working Minds) | Synch  Asynch  N/S | 120 | Members of the workforce | Employees/co-workers of general workplace settings | Videos, role-play/simulation, group discussion, workbook | Information about suicide  Information about being a gatekeeper  Information about resources/referrals |
| Osteen et al [37] | USA | Quantitative; Pre/post evaluation | Law enforcement | Question, Persuade, Refer (QPR) for Law Enforcement | Synch  Asynch  N/S | 90  (+240 – 300 advanced training) | Law enforcement officers | Individual at-risk for suicide | N/S | Information about being a gatekeeper |
| Perepezko et al [38] | N/S | Mixed; Evaluation feasibility usability & efficacy | Online discord servers | Stack Up Overwatch Program (StOP) | Synch  Asynch  N/S | 1920 | Volunteers | Online gaming communities and veterans | N/S | N/S |
| Pilbrow et al [39] | Australia | Mixed; Pre/post evaluation & qualitative content analysis | Pharmacy | Advanced Suicide Prevention Training for Pharmacists | Synch  Asynch  N/S | 150 | Pharmacists | Patients/clients of pharmacies | Video, role-play/simulation, digital workbook, group discussion | Information about suicide  Information about being a gatekeeper  Information about resources/referrals |
| Postuvan et al [40] | Slovenia | Quantitative; Non-randomized controlled trial | N/S | IAlive  (iˇZiv in Slovenian) | Synch  Asynch  N/S | 45-60 | Lay persons | Lay persons | Videos lectures, animated examples, interactive images/graphics with pop-ups | Information about suicide  Information about being a gatekeeper |
| Quinnett., [41] | N/S | Program description | N/S | QPR Pathfinder Training | Synch  Asynch  N/S | 840 – 1200  booster: 2 -10 | General population | Individuals at-risk for suicide | Video, role-play/simulation, reading | Information about suicide  Information about being a gatekeeper  Information about resources/referrals  Information about general mental health |
| Reifegerste et al [42] | Germany | Qualitative; Evaluation, thematic analysis | N/S | Help for relatives | Synch  Asynch  N/S | 80 | People who are worried about a loved one | Men | Videos, audio-recordings, manual, text content | Information about suicide  Information about being a gatekeeper  Information about resources/referrals  Information about general mental health |
| Rein et al [43] | USA | Quantitative; Pre/post evaluation | University | Kognito | Synch  Asynch  N/S | 90 | University students and school staff^5^ | University students | Role-play/simulation | Information about being a gatekeeper |
| Robinson-Link et al [44] | USA | Quantitative; Pre/post evaluation | School | Kognito | Synch  Asynch  N/S | 180 | School staff^5^ | Students | Role-play/simulation | Information about suicide  Information about being a gatekeeper  Information about resources/referrals |
| Roslan et al [45] | Malaysia | Quantitative; Pre/post evaluation | University | Online Advanced C.A.R.E Suicide Prevention Gatekeeper Training Program (AdCARE) | Synch  Asynch  N/S | 180 | Healthcare lecturers | University students | Role-play/simulation | N/S |
| Ross et al [46] | USA | Quantitative; Pre/post evaluation | University | Suicide Prevention for College Student (SPCS) Gatekeepers Program | Synch  Asynch  N/S | 90 | College students | College students | Role-play/simulation, skills practice, discussions, peer cofacilitation | Information about suicide  Information about being a gatekeeper |
| Ross et al [47] |  |  |  |  |  |  |  |  | Role-play/simulation, skills practice, discussions |  |
| Schmeckenbecher et al^6^ [48] | Various | Quantitative; Meta-analysis & systematic review | Various | Various | Synch  Asynch  N/S | 30 – 1200 | Various | N/S | N/S | N/S |
| Seabury [49] | USA | N/A; Program creation description | University | Crisis Counseling: I Am Chipper! | Synch  Asynch  N/S | 120 | Social work university students | Social workers’ clients | Interactive PowerPoint presentation, videos, role-play/simulation, reading material | Information about suicide  Information about being a gatekeeper |
|  |  |  |  | Suicide Assessment: Rube Farmer |  |  |  |  |  |  |
| Seabury [50] | N/S | Quantitative; Quasi-experimental design | University | Crisis Counseling: I Am Chipper! | Synch  Asynch  N/S | 120 | Social work university students | Social workers’ clients | Interactive PowerPoint presentation, videos, role-play/simulation, reading material, quiz/tests | Information about suicide  Information about being a gatekeeper |
|  |  |  |  | Suicide Assessment: Rube Farmer |  |  |  |  |  |  |
| ShantaBridges et al [51] | USA | Quantitative; Pre/post evaluation | University | Suicide Prevention and Awareness for Depression (SPAD) | Synch  Asynch  N/S | 4 weeks | University students | University students | N/S | Information about suicide  Information about being a gatekeeper  Information about resources/referrals |
| Smith-Millman et al [52] | USA | Quantitative; Pre/post evaluation | University | Kognito | Synch  Asynch  N/S | 90 - 120 | University students | University students | Role-play/simulation | Information about being a gatekeeper  Information about resources/referrals |
| Stone et al [53] | USA, Mexico, Australia, Canada, Belgium, New Zealand and Japan | Quantitative; Pre/post evaluation | N/S | Youth suicide prevention: An introduction to gatekeeping | Synch  Asynch  N/S | 480 - 1440 | Coalition members, public health practitioners, school staff^5^, healthcare providers/clinicians | Youth | PowerPoint presentation, quiz/tests, resources, worksheet, audio files | Information about suicide  Information about being a gatekeeper  Information about resources/referrals  Information about general mental health |
| Stover et al [54] | USA | Co-design feasibility study | Community pharmacy | Pharm‐SAVES | Synch  Asynch  N/S | 30 | Pharmacy staff | Pharmacy patients and clients | Videos, reading material, resources | Information about suicide  Information about being a gatekeeper  Information about resources/referrals |
| Sun et al [55] | China | Quantitative; Pre/post evaluation | Family environment | Chinese Life Gatekeeper Training Program (LGTP) | Synch  Asynch  N/S | N/S | Caregivers or legal guardians of adolescents | Adolescents | Videos, role-play/simulation, contextual understanding, group discussion, Q&A session | Information about suicide  Information about being a gatekeeper  Information about resources/referrals |
| Teo et al [56] | USA | Mixed; RCT & Qualitative content analysis | Military | VA S.A.V.E. (Signs; Ask; Validate; Encourage/Expedite) | Synch  Asynch  N/S | 24 | Veterans’ friends and family members | Veterans | Videos | Information about suicide  Information about resources/referrals |
| Teo et al [57] | USA | Mixed; RCT & Qualitative content analysis | Military | VA S.A.V.E. (Signs; Ask; Validate; Encourage/Expedite) | Synch  Asynch  N/S | 25 | Individuals in contact with veterans | Veterans | Videos, vignettes | Information about suicide  Information about being a gatekeeper  Information about resources/referrals |
| Timmons-Mitchell et al [58] | USA | Quantitative; Pre/post evaluation | School | Kognito At-Risk for Middle School Educators | Synch  Asynch  N/S | 45 – 90 | School staff^5^ | Students | Role-play/simulation | Information about suicide  Information about being a gatekeeper  Information about resources/referrals |
| Wislocki et al [59] | N/A | Quantitative; Scoping review | Various (schools, military, religious & tribal communities) | Multiple (n=506)^7^ | Synch  Asynch  N/S | > 2 (M: 44.6; SD: 33.03; R: 2-175) | Gatekeepers or nonprofessional providers (73.1%) including educators, school personnel, caregivers, military personnel, students, employers, etc.; and clinical providers (26.9%)^8^ | N/S | Videos | Information about suicide  Information about being a gatekeeper  Information about resources/referrals |

UK: United Kingdom
USA: United States of America
N/S: not specified
Sync: synchronous
Async: asynchronous
RCT: randomized controlled trial
MOOC: Mass open online course
M: mean
SD: standard deviation
R: range
^1^ For reviews, we only extracted data about training programs that met our inclusion criteria.
^2^ We considered all programs indicating that trainees could complete training “at their own pace” as asynchronous.
^3^ We categorized topics in four categories: **1) Information about suicide** (including information about suicide prevention, suicidal or self-injury behaviors, suicide myths, suicide prevalence and statistics, risk factors for suicide, protective factors against suicide, signs of mental distress/suicidal ideation/warning signs), **2) Information about being a gatekeeper** (including intervention skills and identification of at-risk individuals), **3) Information about resources and referrals,** and **4) Information about general mental health** (including mental fitness, self-care).
^4^ We exclusively reported the topics explicitly mentioned in the article, but we acknowledge that the training programs might cover additional topics not mentioned in the article.
^5^School staff can include teachers, educators, academic advisors, faculty members or general school staff.
^6^ This systematic review and meta-analysis is included for thoroughness; however, data from the primary studies were not re-extracted in this table since most were already included individually, and the remaining did not meet our eligibility criteria.
^7^ This scoping review included 506 training videos. For the sake of conciseness, we did not include the program names in this table.
^8^ Only information regarding training for non-clinical providers was extracted and included in this table.

**References**

1. Afsharnejad B, Milbourn B, Hayden-Evans M, Baker-Young E, Black MH, Thompson C, et al. The efficacy of the “Talk-to-Me” suicide prevention and mental health education program for tertiary students: a crossover randomised control trial. European Child & Adolescent Psychiatry. 2023 2023/12/01;32(12):2477-89. doi: 10.1007/s00787-022-02094-4.

2. Albritton T, Ford KL, Elsbernd K, Santodomingo M, Juzang I, Weddington P, et al. Implementing a Peer Advocate Mental Health Digital Intervention Program for Ohio Youth: Descriptive Pilot Study. JMIR Ment Health. 2021 2021/4/23;8(4):e24605. doi: 10.2196/24605.

3. Bartgis J, Albright G. Online role-play simulations with emotionally responsive avatars for the early detection of native youth psychological distress, inclusing depression and suicidal ideation. American Indian & Alaska Native Mental Health Research: The Journal of the National Center. 2016 06//;23(2):1-27. PMID: 116161795. doi: 10.5820/aian.2302.2016.1.

4. Brown K, Toombs M, Nasir B, Kisely S, Ranmuthugala G, Brennan-Olsen SL, et al. How can mobile applications support suicide prevention gatekeepers in Australian Indigenous communities? Social Science & Medicine. 2020 2020/08/01/;258:113015. doi: 10.1016/j.socscimed.2020.113015.

5. Bryant R, Vogt M, Miller C. Effects of an Avatar-Based Simulation on Family Nurse Practitioner Students’ Self-Evaluated Suicide Prevention Knowledge and Confidence. Nursing Education Perspectives. 2025;46(3). doi: 10.1097/01.NEP.0000000000001321.

6. Canady VA. National, local efforts train educators on suicide prevention, MH support. Mental Health Weekly. 2015;25(22):1-7. doi: 10.1002/mhw.30206.

7. Carpenter DM, Roberts CA, Lavigne JE, Cross WF. Gatekeeper training needs of community pharmacy staff. Suicide and Life-Threatening Behavior. 2021;51(2):220-8. doi: 10.1111/sltb.12697.

8. Caughlan C, Kakuska A, Manthei J, Galvin L, Martinez A, Kelley A, et al. Mind4Health: decolonizing gatekeeper trainings using a culturally relevant text message intervention. Frontiers in Public Health. 2024 2024-September-02;Volume 12 - 2024. doi: 10.3389/fpubh.2024.1397640.

9. Cohen E, Pomerance Y, Touati Ohayon L, Brunstein Klomek A. Efficacy of suicide prevention gatekeeper training in Israel: Exploring diverse at-risk populations, gender differences, and comparisons between online and in-person training. Death Studies. 2025:1-9. doi: 10.1080/07481187.2025.2510482.

10. Colder Carras M, Bergendahl M, Labrique AB. Community Case Study: Stack Up’s Overwatch Program, an Online Suicide Prevention and Peer Support Program for Video Gamers. Frontiers in Psychology. 2021 2021-March-11;Volume 12 - 2021. doi: 10.3389/fpsyg.2021.575224.

11. Coleman D, Black N, Ng J, Blumenthal E. Kognito's Avatar-Based Suicide Prevention Training for College Students: Results of a Randomized Controlled Trial and a Naturalistic Evaluation. Suicide and Life-Threatening Behavior. 2019;49(6):1735-45. doi: 10.1111/sltb.12550.

12. Colucci E, Sirar J, and Rossmann M. Piloting of a suicide first aid gatekeeper training (online) for children and young people in conflict affected areas in Syria. International Review of Psychiatry. 2022 2022/08/18;34(6):640-8. doi: 10.1080/09540261.2022.2100245.

13. Ghoncheh R, Gould MS, Twisk JW, Kerkhof AJ, Koot HM. Efficacy of Adolescent Suicide Prevention E-Learning Modules for Gatekeepers: A Randomized Controlled Trial. JMIR Mental Health. 2016;3(1):e8. PMID: 26825006. doi: 10.2196/mental.4614.

14. Ghoncheh R, Koot HM, Kerkhof AJ. Suicide prevention e-learning modules designed for gatekeepers: a descriptive review. Crisis. 2014;35(3):176-85. PMID: 24901058. doi: 10.1027/0227-5910/a000249.

15. Hawley SR, Skinner T, Young M, St Romain T, Provines J. Suicide Prevention Across the Community: Evaluation of Mental Health Training for Multiple Gatekeeper Groups. Kans J Med. 2024 Nov-Dec;17(6):127-32. PMID: 39758535. doi: 10.17161/kjm.vol17.22524.

16. Hill RM, and McCray CL. Suicide-Related Stigma and Social Responsibility Moderate the Effects of an Online Suicide Prevention Gatekeeper Training Program. Archives of Suicide Research. 2023 2024/04/02;28(2):706-15. doi: 10.1080/13811118.2023.2199802.

17. Hill RM, Picou P, Hussain Z, Vieyra BA, Perkins KM. Randomized Controlled Trial of an Online Suicide Prevention Gatekeeper Training Program: Hogrefe Publishing; 2024. 57–64 p. ISBN: 0227-5910.

18. Hofmann L, Glaesmer H, Przyrembel M, Wagner B. An Evaluation of a Suicide Prevention E-Learning Program for Police Officers (COPS): Improvement in Knowledge and Competence. Frontiers in Psychiatry. 2021 2021-December-13;Volume 12 - 2021. doi: 10.3389/fpsyt.2021.770277.

19. Hofmann L, Wagner B. Efficacy of an online gatekeeper program for relatives of men at risk of suicide – a randomized controlled trial. BMC Public Health. 2024 2024/10/02;24(1):2693. doi: 10.1186/s12889-024-20193-6.

20. Holmes G, Amanda C, Anita H, and Kõlves K. Online versus in-person gatekeeper suicide prevention training: comparison in a community sample. Journal of Mental Health. 2024 2024/09/02;33(5):605-12. doi: 10.1080/09638237.2024.2332811.

21. Kawashima D, Yoshiki K, and Yoshioka M. Feasibility of brief online gatekeeper training for Japanese university students: A randomized controlled trial. Death Studies. 2023 2023/05/28;47(5):531-40. doi: 10.1080/07481187.2022.2101076.

22. Kimbrel NA, Aho NA, Neal LC, Bernes SA, Beaver TA, Hertzberg JA, et al. Development and Implementation of Web-Based Safety Planning Intervention Training for Firefighter Peer Support Specialists. Crisis. 2024;45(2):108-17. PMID: 37727969. doi: 10.1027/0227-5910/a000924.

23. Kingi-Ulu'ave D, Framptom C, Cargo T, Stasiak K, Hetrick S. Evaluating the effectiveness of a post-training enhancement to the LifeKeepers suicide prevention gatekeeper training: A randomized controlled trial. Crisis: The Journal of Crisis Intervention and Suicide Prevention. 2025;46(3):157-65. doi: 10.1027/0227-5910/a001001.

24. Kingi-Uluave D, Taufa N, Tuesday R, Cargo T, Stasiak K, Merry S, et al. A Review of Systematic Reviews: Gatekeeper Training for Suicide Prevention with a Focus on Effectiveness and Findings. Archives of Suicide Research. 2025 2025/04/03;29(2):329-46. doi: 10.1080/13811118.2024.2358411.

25. Kreuze E, Ruggiero KJ. Technology-Oriented Suicide Prevention Interventions for Adolescents and Adolescent Gatekeepers: A Qualitative Review. Adolescent Research Review. 2018 2018/06/01;3(2):219-33. doi: 10.1007/s40894-017-0060-5.

26. Kreuze E, York J, Lamis DA, Jenkins C, Quinnett P, Mueller M, et al. Gatekeeper training for youth suicide prevention: A mixed method comparative analysis of two online programs. Psychology in the Schools. 2025;62(2):492-511. doi: 10.1002/pits.23335.

27. Lamis DA, Underwood M, D'Amore N. Outcomes of a Suicide Prevention Gatekeeper Training Program Among School Personnel. Crisis. 2017;38(2):89-99. PMID: 27561223. doi: 10.1027/0227-5910/a000414.

28. Lancaster PG, Moore JT, Putter SE, Chen PY, Cigularov KP, Baker A, et al. Feasibility of a Web-based Gatekeeper Training: Implications for Suicide Prevention. Suicide and Life-Threatening Behavior. 2014;44(5):510-23. doi: 10.1111/sltb.12086.

29. Lee-Tauler SY, Grammer J, LaCroix JM, Walsh AK, Clark SE, Holloway KJ, et al. Pilot Evaluation of the Online ‘Chaplains-CARE’ Program: Enhancing Skills for United States Military Suicide Intervention Practices and Care. Journal of Religion and Health. 2023 2023/12/01;62(6):3856-73. doi: 10.1007/s10943-023-01882-9.

30. Liu H, Zheng C, Cao Y, Zeng F, Chen H, Gao W. Gatekeeper training for suicide prevention: a systematic review and meta-analysis of randomized controlled trials. BMC Public Health. 2025 2025/03/31;25(1):1206. doi: 10.1186/s12889-025-21736-1.

31. MacDonald Hart S, Colucci E, Marzano L. Evaluating suicide prevention gatekeeper training designed to identify and support people from asylum-seeking and refugee backgrounds. BMC Public Health. 2024;24. doi: 10.1186/s12889-024-20304-3.

32. Manning J, and VanDeusen K. Suicide Prevention in the Dot Com Era: Technological Aspects of a University Suicide Prevention Program. Journal of American College Health. 2011 2011/04/08;59(5):431-3. doi: 10.1080/07448480903540507.

33. Marley G, Lavigne JE, Cross W, Gamble A, Zhang Z, Carpenter DM. Comparing three methods to assess learning outcomes for a suicide prevention training program for pharmacy staff. PEC Innovation. 2024 2024/12/15/;5:100348. doi: 10.1016/j.pecinn.2024.100348.

34. McKay S, Byrne SJ, Clarke A, Lamblin M, Veresova M, Robinson J. Parent Education for Responding to and Supporting Youth with Suicidal Thoughts (PERSYST): An Evaluation of an Online Gatekeeper Training Program with Australian Parents. International Journal of Environmental Research and Public Health. 2022;19(9):5025. PMID: doi:10.3390/ijerph19095025. doi: 10.3390/ijerph19095025.

35. Mirick RG. Acceptability and Feasibility of a Brief Online Suicide Prevention Training for School Staff. Journal of Technology in Human Services. 2025 2025/01/02;43(1):34-48. doi: 10.1080/15228835.2024.2447692.

36. Mishkind MC, Yannacone A, Lopez A, Jortberg BT, Sherrill A, Mescher T. Virtual Versus In-Person Suicide Prevention Training in the Workplace: Evaluation of the VitalCog Program. Journal of Technology in Behavioral Science. 2023 2023/12/01;8(4):352-9. doi: 10.1007/s41347-023-00301-w.

37. Osteen PJ, Ohme K, Morris RC, Arciniegas J, Frey JJ, Woods M, et al. Suicide intervention training with law enforcement officers. Suicide and Life-Threatening Behavior. 2021;51(4):785-94. doi: 10.1111/sltb.12763.

38. Perepezko K, Bergendahl M, Kunz C, Labrique A, Carras M, Colder Carras M. “Instead, You’re Going to a Friend”: Evaluation of a Community-Developed, Peer-Delivered Online Crisis Prevention Intervention. Psychiatric Services. 2024 2024/12/01;75(12):1267-75. doi: 10.1176/appi.ps.20230233.

39. Pilbrow S, Staniland L, Uren HV, Shand F, McGoldrick J, Thorp E, et al. Evaluation of an online advanced suicide prevention training for pharmacists. International Journal of Clinical Pharmacy. 2023 2023/10/01;45(5):1203-11. doi: 10.1007/s11096-023-01636-3.

40. Poštuvan V, Gomboc V, Čopič Pucihar K, Kljun M, Vičič J, Tančič Grum A, et al. Development and Evaluation of Online Suicide Preventive Tool iAlive to Increase Competences in Engaging With a Suicidal Person. Crisis. 2023 2024/05/01;45(3):187-96. doi: 10.1027/0227-5910/a000934.

41. Quinnett PG. The Certified QPR Pathfinder Training Program: A Description of a Novel Public Health Gatekeeper Training Program to Mitigate Suicidal Ideation and Suicide Deaths. Journal of Prevention. 2023 2023/12/01;44(6):813-24. doi: 10.1007/s10935-023-00748-w.

42. Reifegerste D, Wagner AJM, Huber L, Fastuca M. Formative Evaluation of Suicide Prevention Websites for Men: Qualitative Study with Men at Risk of Suicide and with Potential Gatekeepers. JMIR Form Res. 2025 2025/2/26;9:e59829. doi: 10.2196/59829.

43. Rein BA, McNeil DW, Hayes AR, Hawkins TA, Ng HM, Yura CA. Evaluation of an avatar-based training program to promote suicide prevention awareness in a college setting. J Am Coll Health. 2018;66(5):401-411. [doi: 10.1080/07448481.2018.1432626] [Medline: 29461940]

44. Robinson-Link N, Hoover S, Bernstein L, Lever N, Maton K, Wilcox H. Is Gatekeeper Training Enough for Suicide Prevention? School Mental Health. 2020 2020/06/01;12(2):239-49. doi: 10.1007/s12310-019-09345-x.

45. Roslan AF, Pheh KS, Mahadevan R, Bujang SM, Subramaniam P, Yahya HF, et al. Effectiveness of online advanced C.A.R.E suicide prevention gatekeeper training program among healthcare lecturers and workers in national university of Malaysia: A pilot study. Frontiers in Psychiatry. 2023 2023-January-19;Volume 14 - 2023. doi: 10.3389/fpsyt.2023.1009754.

46. Ross SG, Pazienza R, Rosa JD. The Suicide Prevention for College Student (SPCS) Gatekeepers. Program: A 3-Year Review of the Evidence. 2024;45(1):41–7. doi: 10.1027/0227-5910/a000914.

47. Ross SG, Pazienza R, Rosa JD. The Suicide Prevention for College Students (SPCS) Gatekeepers Program: Comparing in-person and online training outcomes. Journal of American College Health. 2024:1-4. doi: 10.1080/07448481.2024.2423237.

48. Schmeckenbecher J, Lentner S, Emilian CA, Plener PL, Baran A, Kapusta ND. E-learning as a tool of suicide prevention training: A meta-analysis and systematic review. Death Studies. 2024 2024/10/20;48(9):962-74. doi: 10.1080/07481187.2023.2297058.

49. Seabury B. On-Line, Computer-Based, Interactive Simulations: Bridging Classroom and Field. Journal of Technology in Human Services. 2003 2003/12/08;22(1):29-48. doi: 10.1300/J017v22n01_04.

50. Seabury BA. An Evaluation of On-Line, Interactive Tutorials Designed to Teach Practice Concepts. Journal of Teaching in Social Work. 2005 2005/08/01;25(1-2):103-15. doi: 10.1300/J067v25n01_07.

51. Shanta Bridges L, Sharma M, Lee JHS, Bennett R, Buxbaum SG, Reese-Smith J. Using the PRECEDE-PROCEED model for an online peer-to-peer suicide prevention and awareness for depression (SPAD) intervention among African American college students: experimental study. Health Promot Perspect. 2018;8(1):15-24. PMID: 29423358. doi: 10.15171/hpp.2018.02.

52. Smith-Millman M, Larraine B, Natasha L, Sharon H, and Lever N. Effectiveness of an online suicide prevention program for college faculty and students. Journal of American College Health. 2022 2022/06/22;70(5):1457-64. doi: 10.1080/07448481.2020.1804389.

53. Stone DM, Barber CW, Potter L. Public Health Training Online: The National Center for Suicide Prevention Training. American Journal of Preventive Medicine. 2005 2005/12/01/;29(5, Supplement 2):247-51. doi: 10.1016/j.amepre.2005.08.019.

54. Stover AN, Lavigne JE, Shook A, MacAllister C, Cross WF, Carpenter DM. Development of the Pharm-SAVES educational module for gatekeeper suicide prevention training for community pharmacy staff. Health Expectations. 2023;26(3):1246-54. doi: 10.1111/hex.13741.

55. Sun Y, Zhang Q, Wu W, Lin J, Sun S, An J, et al. Efficacy of a localized caregiver gatekeeper training program for suicide prevention among Chinese adolescents: A pilot study. Asian Journal of Psychiatry. 2025 2025/07/01/;109:104555. doi: 10.1016/j.ajp.2025.104555.

56. Teo AR, Call AA, Hooker ER, Fong C, Karras E, Dobscha SK. Feasibility of recruitment and retention in a remote trial of gatekeeper training for close supports of military veterans: Mixed methods study. Contemporary Clinical Trials Communications. 2022 2022/12/01/;30:100993. doi: 10.1016/j.conctc.2022.100993.

57. Teo AR, Hooker ER, Call AA, Dobscha SK, Gamble S, Cross WF, et al. Brief video training for suicide prevention in veterans: A randomized controlled trial of VA S.A.V.E. Suicide and Life-Threatening Behavior. 2024 2024/02/01;54(1):154-66. doi: 10.1111/sltb.13028.

58. Timmons-Mitchell J, Albright G, McMillan J, Shockley K, Cho S. Virtual role-play: middle school educators addressing student mental health. Health Behavior and Policy Review. 2019;6(6):546-57. doi: 10.14485/HBPR.6.6.1.

59. Wislocki K, Jager-Hyman S, Brady M, Weiss M, Schaechter T, Khazanov G, et al. Freely Available Training Videos for Suicide Prevention: Scoping Review. JMIR Ment Health. 2023 2023/11/3;10:e48404. doi: 10.2196/48404.
