# Supplementary material for: Key Components and Barriers in Web-Based Suicide Prevention Gatekeeper Training: Systematic Narrative Review
Source: J Med Internet Res. 2026 Feb 5;28:e81572. doi: 10.2196/81572 (PMC12921433; doi:10.2196/81572)
Supplement: Multimedia Appendix 4 [file jmir_v28i1e81572_app4.docx]

| **Domain** | **Do’s (Recommended Practices)** | **Don’ts (Practices to Avoid)** |
| --- | --- | --- |
| **Design** | - Ground GTPs in theory and evidence-based practices - Align and integrate with existing suicide prevention initiatives - Use co-design approaches with stakeholders and end users - Ensure adequate resources for sustainability (moderation, maintenance, updates, improvements) - Minimize technical barriers (low bandwidth, program glitches) - Provide multi-device compatibility and multiple formats (audio, video, text, captions) - Keep training brief but aligned with learning objectives - Ensure affordability of GTP - Customize content to learner needs, backgrounds, and experience - Offer flexible pacing and segmented sessions - Incorporate mechanisms for standardization (e.g., automated feedback, pre-recorded videos) - Safeguard privacy (secure data storage, limit third-party risks). | - Treat GTP as a standalone solution disconnected from broader suicide prevention initiatives - Overlook accessibility challenges (e.g., bandwidth, disabilities) - Use overly long sessions that discourage participation - Provide one-size-fits-all content without customization - Rely solely on instructor expertise for consistency - Use unsecured third-party platforms without safeguards - Neglect long-term sustainability planning |
| **Content** | - Use clear, simple, non-clinical language - Normalize discussion of suicide in non-stigmatizing ways - Cover essential topics: suicide knowledge (stats, myths, risk factors), gatekeeper skills (warning signs, conversation steps, safety planning), referral guidance, gatekeeper well-being and self-care - Tailor content to trainee backgrounds, contexts, and cultures - Include diverse and inclusive examples - Emphasize interpersonal and motivational skills (e.g., active listening, compassion, open-ended questions) - Address advocacy for marginalized populations as a gatekeeping skill | - Use technical jargon or clinical terms - Include graphic details of suicide deaths - Present irrelevant or overly procedural content (e.g., professional reporting not applicable to trainees) - Overlook cultural adaptation or diversity in examples |
| **Pedagogy** | - Incorporate interactive learning (role-plays, scenarios, practice exercises) - Use innovative technologies (e.g., avatars for simulations) - Include knowledge checks (tests, quizzes) with immediate feedback - Allow self-paced learning while offering guidance and reminders - Provide motivational supports (reminders, incentives) - Build communities of practice (forums, peer buddies, moderated groups, virtual coaches) - Offer reinforcement strategies (refreshers, follow-ups, digital workbooks) | - Rely exclusively on didactic, non-interactive methods - Overestimate trainees’ intrinsic motivation without support - Ignore the need for feedback or guidance - Leave learners isolated without peer or instructor interaction - Overuse repetition to the point of distraction |
